# The macrophage galactose‐type C‐type lectin 1 receptor plays a major role in mediating colitis‐associated colorectal cancer malignancy

**DOI:** 10.1111/imcb.70011

**Published:** 2025-03-03

**Authors:** Oscar Nieto‐Yañez, Sonia H Navia, Imelda Juárez‐Avelar, Tonathiu Rodríguez, Antonio Andrade‐Meza, Betsaida J Ortiz‐Sánchez, Mónica G Mendoza‐Rodríguez, Jonadab E Olguín, José L Reyes, Daniel Montes de Oca‐Samperio, Citlaltepetl Salinas Lara, Luis I Terrazas, Miriam Rodriguez‐Sosa

**Affiliations:** ^1^ Innate Immunity Laboratory, Biomedicine Research Unit (UBIMED), Facultad de Estudios Superiores Iztacala (FES‐Iztacala) Universidad Nacional Autónoma de México (UNAM) Tlalnepantla State of Mexico Mexico; ^2^ Doctoral Program in Biological Sciences, UNAM Mexico City Mexico; ^3^ Undergraduate Program of Optometry, FES‐Iztacala, UNAM Tlalnepantla Mexico; ^4^ Doctoral Program in Biomedical Sciences, UNAM Mexico City Mexico; ^5^ Undergraduate Program of Dental Surgeon, FES‐Iztacala, UNAM Tlalnepantla Mexico; ^6^ Immunoparasitology Laboratory, UBIMED, FES‐Iztacala, UNAM Tlalnepantla Mexico; ^7^ Flow Cytometry Area, National Laboratory in Health: Molecular Diagnosis and Environmental Effect on Chronic Degenerative Diseases, FES‐Iztacala, UNAM Tlalnepantla Mexico; ^8^ Laboratory of Experimental Immunology and Regulation of Gut‐liver Inflammation, UBIMED, FES Iztacala, UNAM Tlalnepantla Mexico; ^9^ MEDICI Network, Molecular Pathogenesis Laboratory, Medical Surgeon Career, FES Iztacala, UNAM Tlalnepantla Mexico

**Keywords:** colorectal cancer, C‐type lectin‐like receptors, macrophage galactose‐C type lectin, mMGL1 receptor, myeloid suppressor cells

## Abstract

Cancer‐associated aberrant glycosylation can be detected by the macrophage galactose‐type C‐type lectin (MGL) receptor; however, whether this interaction enhances or deadens cancer development along with the associated immune response has not been well established. To determine the role of mouse MGL1 in colitis‐associated colon cancer (CAC), azoxymethane (AOM)/dextran sodium sulfate (DSS)‐induced tumor development was compared between *Mgl1* knockout (*Mgl1* ^−/−^) mice and their wild‐type (WT) littermates. At 75 days post‐CAC induction, colon tumor tissue contained more highly glycosylated proteins, representing potential ligands for the mMGL1 receptor, than did healthy colon tissue. The *Mgl1* ^*−/−*^ CAC mice scored lower in disease activity indices and had fewer colonic tumors. In addition, the colonic crypt architecture was less damaged, and mucin production was more significant than in the WT CAC mice. Furthermore, *Mgl1* ^*−/−*^ CAC mice displayed higher percentages of CD4^+^ and CD8^+^ T cells in the peripheral blood, and colonic lamina propria; and lower percentages of myeloid‐derived suppressor cells (MDSCs). Additionally, less macrophage (Mφ) and natural killer (NK) cell infiltration and lower levels of iNOS and arginase were found in the tumor microenvironment of *Mgl1* ^*−/−*^ CAC mice compared with WT mice. These results suggest that the mMGL1 receptor may recognize aberrant glycosylation in colon cancer, which may trigger an inflammatory microenvironment and favor colon tumorigenesis.

## Graphical abstract

In this study, we found that aberrant glycosylation of colitis‐associated colorectal cancer (CAC) can be detected by the mouse macrophage galactose C‐type lectin 1 (MGL). The Mgl1 knockout mice with CAC developed fewer symptoms and smaller tumors, associated with a decrease in monocytic myeloid‐derived suppressor cells and reduced the proinflammatory microenvironment. This suggests that MGL plays a role in downregulation of the immune response, which contributes to the development and malignancy of CAC.

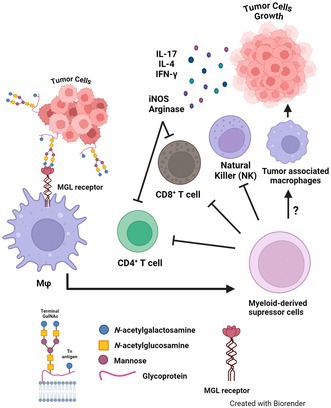

## INTRODUCTION

Colorectal cancer is the third most common cancer worldwide.^1^ According to the GLOBOCAN report, in 2022, 1 926 425 new patients were diagnosed with colorectal carcinoma, and 904 019 died from this disease. Approximately 329 037 cases and 113 577 deaths have been reported in people younger than 54 years.^2^ Colorectal cancer results from complex environmental, immune and genetic interactions. Some of these factors include age, sex, ethnicity, obesity, family history and metabolic and inflammatory bowel diseases (IBDs), which include ulcerative colitis and Crohn's disease.^3^

Colitis‐associated colon cancer (CAC) is closely related to long‐standing IBD, which leads to damage to epithelial cells and dysbiosis. This condition may affect the expression of genes associated with oxidative stress and DNA repair pathways and attract immune cells to detect and eliminate transformed cells. However, reduced detection of transformed cells due to alterations in the immune response leads to increased cell proliferation and tumor growth.^4^

The development of CAC involves interactions between tumor cells and the host microenvironment, which consists of immune cells, fibroblasts, blood, lymphatic vascular endothelial cells and the extracellular matrix.^3^ Among the stromal cell populations infiltrating tumors are myeloid cells, which comprise mainly tumor‐associated macrophages (TAMs), dendritic cells (DCs), tumor‐associated neutrophils (TANs) and myeloid‐derived suppressor cells (MDSCs). These cells mediate cancer initiation, progression and resistance to cancer therapy.^5^

Therefore, crosstalk between cancer cells and immune cells is crucial for promoting or inhibiting tumor growth and metastasis.^6^ This communication between cells is influenced primarily by pattern recognition receptors (PRRs), such as C‐type lectin receptors, among others.^7^ C‐type lectin receptors, such as DC‐SIGN, CD93, CLEC14A, CLEC2, and MMR, are widely expressed on Mφs, neutrophils and DCs. They contain one or more C‐type lectin‐like domains, which are essential for recognizing carbohydrate structures in pathogens and host tissues.^8^

The activation of C‐type lectin receptors by their ligands initiates intracellular signaling pathways that regulate the immune response, including DC maturation, decreased phagocytosis, reactive oxygen species production and inflammasome activation.^9^ Furthermore, some C‐type lectin receptors expressed in afferent and efferent lymphatic vessels promote lymphatic metastasis by interacting with lectins that are expressed in cancer cells.^10^

The macrophage galactose type C‐type lectin (MGL) receptor is a member of the C‐type lectin family, and its expression is associated with Mφs, immature DCs and mast cells in humans and mice.^11^ In mice, there are two MGL orthologs, mouse (m) MGL1 and mMGL2,^12^ although glycan binding studies have demonstrated that both contain the Gln‐Pro‐Asp sequence (QPD) in their carbohydrate recognition domain (CRD).^13^ The sugar‐binding specificity is determined by a combination of factors, including the specific amino acid residues within the CRD and the overall shape and geometry of the binding site.^14^ mMGL1 receptor recognizes Lewis X (GALβ1‐4 (Fucα1‐3) GlcNAc) and Lewis A (Galβ1‐3 (Fucα1‐4) GlcNAc) carbohydrates with high affinity, whereas mMGL2 recognizes carbohydrates containing α‐GalNAc, similar to the carbohydrate specificity of human MGL (hMGL).^15^

Colon tumor cells display antigenic alterations, such as aberrant glycosylation that the human MGL receptor can recognize.^16^, ^17^ For example, human colorectal tumor cell lines display mucin proteins that are highly glycosylated with Tn antigen (MUC1‐Tn), sialylated Tn antigen (sTn) and LacdiNAc. Tn is a glycan structure formed by N‐acetyl‐D‐galactosamine and a glycosidic α‐linkage to serine/threonine residues in glycoproteins (GalNAcα1‐*O*‐Ser/Thr).^18^ Thus, *in vitro*, the hMGL receptor binds with high specificity to terminal α‐and β‐linked GalNAc residues found in Tn, sTn (NeuAcα2,6GalNAcα1‐Ser/Thr) and LacdiNAc (GalNAcβ1,4GlcNAcβ1‐).^19^

Moreover, tumors from patients with colorectal cancer with mutations in the BRAF gene are highly positive for glycosylated MGL ligands, which correlates with poor survival.^20^ Therefore, the profile of carbohydrates present in colon tumors potentially plays a role in tumor immunity and/or development. Thus, the role of glycosylation and its interaction with MGL in the colon tumor microenvironment needs to be defined.

In the present study, using *Mgl1* ^*−/−*^ mice, we found that the mMGL1 receptor plays a role in favoring colon tumorigenesis.

## RESULTS

### The mMGL1 receptor is expressed in wild‐type macrophages and dendritic cells, and its ligands are upregulated during AOM/DSS‐induced cancer

To validate the deletion of mMGL1, we analyzed mMGL1 and mMGL2 expression in bone marrow‐derived DCs and Mφs from WT and *mMgl1* ^*−/−*^ mice by flow cytometry.

**Figure 1:**
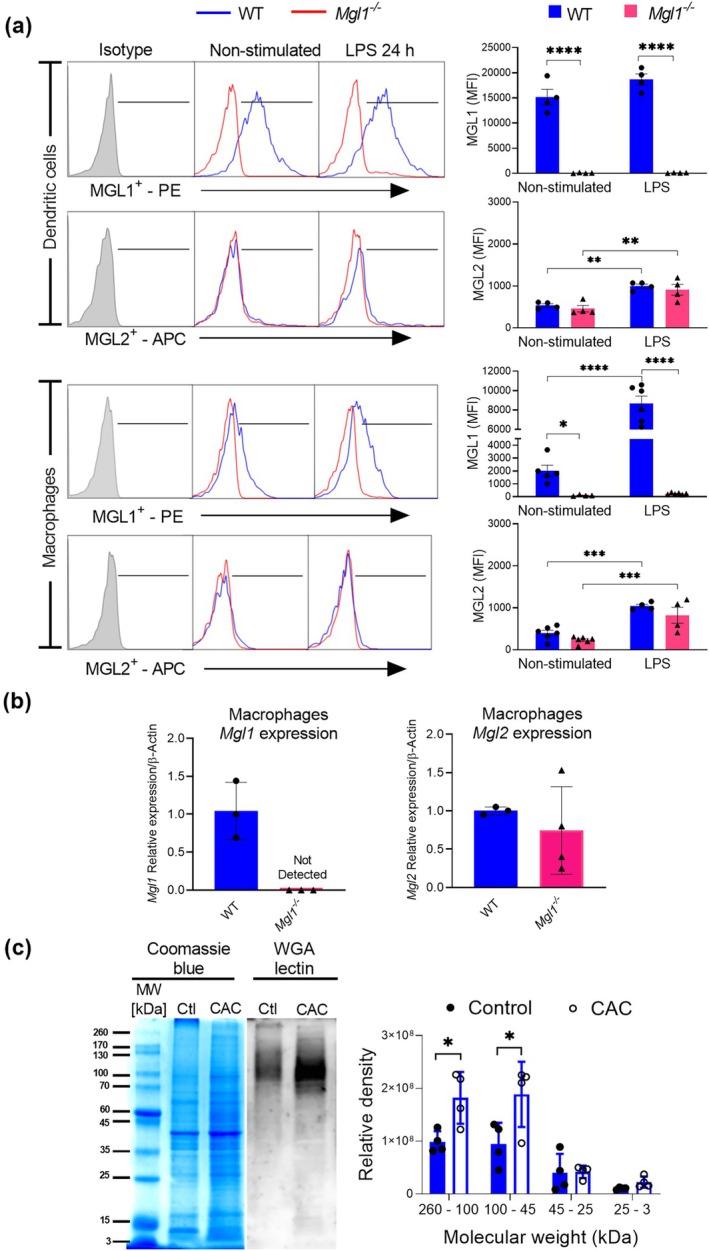
The mMGL1 receptor is not expressed in dendritic cells or macrophages from *Mgl1* ^*−/−*^ mice, and total CAC tumor antigens are rich in glycoproteins. **(a)** Representative histogram and bar graphs of mMGL1 and mMGL2 expression in bone marrow‐derived dendritic cells and macrophages from healthy control WT and *Mgl1* ^*−/−*^ mice without or with LPS (100 ng mL^−1^) stimulation. **(b)** *Mgl1* and *Mgl2* mRNA expression in bone marrow‐derived macrophages from healthy control mice was determined by qRT‐PCR and normalized to β actin mRNA expression. **(c)** Representative image of the relative density of the protein bands stained with wheat germ agglutinin lectin (WGA). Distal colon proteins from healthy control and CAC WT mice were separated by a gradient 4–20% SDS‐PAGE gel and transferred to a PVDF (polyvinylidene difluoride) membrane. The SDS–PAGE gels were stained with Coomassie blue, and the membranes were incubated with WGA lectin conjugated to horseradish peroxidase (HRP). The signal was revealed using SuperSignal West Femto and then digitalized using a UVITEC Cambridge Chemiluminescence Imaging System and analyzed using the Image Lab Software Version 6.1.0 build 7 Standard Edition from Bio‐Rad Laboratories. The data are representative of three independent experiments and are presented as the mean ± standard error, *n* = 3–6 animals. Statistical significance was determined by one‐way ANOVA or Student's *t*‐tests, paired or unpaired as appropriate, with GraphPad Prism 8.3 software. *P < 0.05; **P < 0.01; ***P < 0.00; ****P < 0.0001.

As shown in Figure 1a, unstimulated WT DCs exhibited basal levels of MGL1 expression, whereas this expression was undetectable in *Mgl1* ^*−/−*^ DCs (WT nonstimulated vs. *Mgl1* ^*−/−*^ nonstimulated, ****P < 0.0001). MGL1 expression increased slightly after WT DCs stimulation with LPS, but MGL1 expression was undetectable in LPS‐primed DCs from *Mgl1* ^*−/−*^ mice (WT LPS vs. *Mgl1* ^*−/−*^ LPS ****P < 0.0001).

Importantly, DCs from both WT and *Mgl1* ^*−/−*^ mice exhibited similar basal mMGL2 expression levels, which increased significantly with LPS stimulation (Figure 1a, WT nonstimulated vs. WT LPS; *Mgl1* ^*−/−*^ nonstimulated vs. *Mgl1* ^*−/−*^ LPS; **P < 0.01). No significant differences in mMGL2 expression were detected between DCs from WT and *Mgl1* ^*−/−*^ mice stimulated with or without LPS (Figure 1a, MGL2).

Moreover, nonstimulated WT Mφs presented basal mMGL1 expression, which increased significantly in response to LPS (Figure 1a, WT nonstimulated vs. WT LPS, ****P < 0.0001). mMGL1 expression was absent in unstimulated Mφs *Mgl1* ^*−/−*^ (Figure 1a, WT not stimulated vs. *Mgl1* ^−/−^ not stimulated, *P < 0.05), as was the case in Mφs *Mgl1* ^*−/−*^ LPS (WT LPS vs. *Mgl1* ^*−/−*^ LPS, ****P < 0.0001). Mφs from WT and *Mgl1* ^*−/−*^ mice exhibited basal mMGL2 expression, and no significant differences in mMGL2 expression were detected in response to LPS stimulation (Figure 1a, MGL2). *mMgl1* and *mMgl2* mRNA expression was confirmed by RT–PCR in bone marrow‐derived macrophages from control mice. No mMGL1 expression was detected in the *Mgl1* ^*−/*−^ mice, whereas mMGL2 was expressed in the WT and *Mgl1* ^−/−^ mice. These data confirm that the genetically targeted deletion of mMGL1 is highly specific and that mMGL2 expression in DCs and Mφs does not depend on mMGL1 expression.

Next, a wheat germ agglutinin (WGA) lectin blot analysis was performed on total protein from colons of WT mice with or without CAC to confirm that colon tumors generated by chemical induction of CAC contain ligands for the mMGL1 receptor. WGA was used on the basis of a previous report indicating that this lectin binds specifically to N‐acetylglucosamine (GlcNac) in the context of Galβ1‐4GlcNAc, a component of the Lewis X structure.^21^

Lectin blot analysis revealed that total tumor antigens generated by the CAC model contained more high‐molecular‐weight glycoproteins (above 60 kDa) that are strongly glycosylated than healthy control colon tissue. Proteins with a molecular weight less than 45 kDa presented very poor glycosylation, given that no strong signals were detected in this area of the lectin blot (Figure 1c). This result shows that tumors in the CAC mouse model display an altered glycosylation profile in which high‐molecular‐weight glycoproteins are strongly glycosylated, which is similar to findings in human colon cancer tissue.^22^

### Mgl1^−/−^ mice display a lower disease activity index than WT mice after AOM/DSS‐induced colon carcinogenesis

To determine the influence of mMGL1 receptor on colorectal cancer development, we evaluated the effect of *mMgl1* deletion on tumor development in the AOM/DSS model in *Mgl1* ^*−/−*^ and WT mice. Body weight, diarrhea and fecal blood were recorded weekly until day 75 postinduction.

**Figure 2:**
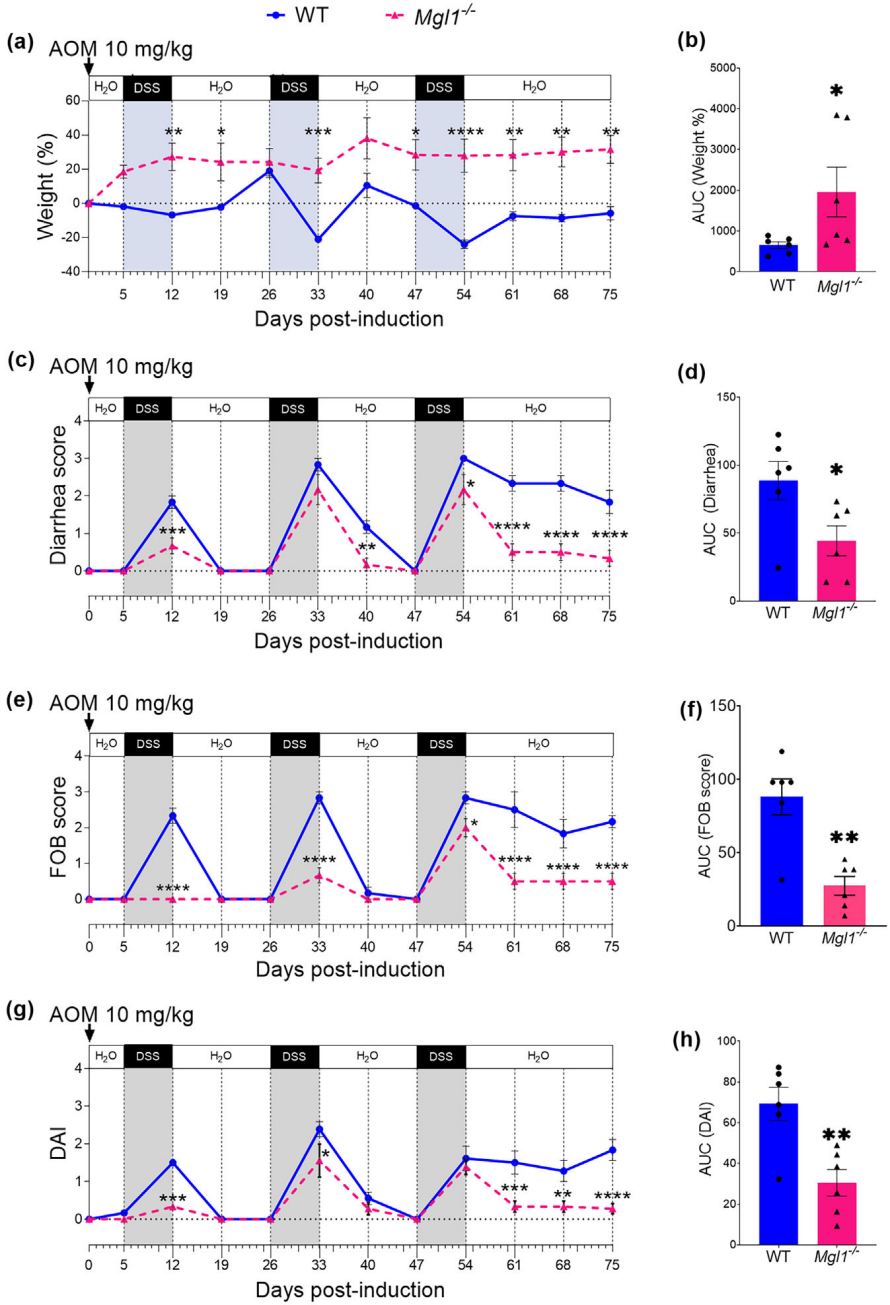
mMGL1 deficiency reduces the incidence of clinical signs of colitis‐associated colon cancer. The disease activity index (DAI) of the WT and *Mgl1* ^*−/−*^ mice were monitored during 75 days of chemical CAC induction with azoxymethane (AOM) and dextran sodium sulfate (DSS). **(a)** Weight (%), **(c)** diarrhea, **(e)** fecal occult blood (FOB) score, **(g)** DAI score. Bar graphs represent the area under the curve (AUC) for **(b)** weight, **(d)** diarrhea, **(f)** FOB scores and **(h)** DAI scores. The DAI data represent three independent experiments plotted as the mean ± standard error. The AUC represents the average individual data for mice, *n* = 6. The statistical significance of the differences between *Mgl1* ^*−/−*^ CAC and WT CAC was determined using two‐way ANOVA in **a, c, e, g**. Student's paired *t*‐test with Welch's correction in **b, d, f, h**; *P < 0.05, **P < 0.01, ***P < 0.001, ****P < 0.0001. GraphPad Prism 8.3 software was used.

Compared with WT mice, *Mgl1* ^*−/−*^ mice displayed significantly attenuated weight loss over the experimental period. WT mice had significant weight loss compared with *Mgl1* ^*−/−*^ mice (Figure 2a; **P < 0.01, ****P < 0.0001, respectively) at the end of the second and third DSS cycles. Significantly, *Mgl1* ^*−/−*^ mice weighed more at 54, 61, 68 and 75 days than WT mice (Figure 2a, **P < 0.01). The average area under the curve (AUC), obtained with data from each mouse, confirmed that *Mgl1* ^*−/−*^ mice gained weight, whereas WT CAC mice did not (Figure 2b, *P < 0. 05).

In accordance with weight differences, the *Mgl1* ^*−/−*^ mice had less diarrhea at 12, 40, 54, 61, 68 and 75 days post‐AOM treatment than did the WT mice (Figure 2c; day 12 ***P < 0.001, day 40 **P < 0.01, day 54 *P < 0.05; and days 61, 68 and 75 ****P < 0.0001). AUC analysis revealed that the *Mgl1* ^*−/−*^ mice presented significantly less diarrhea than the WT mice during the entire CAC development period (Figure 2d, *P < 0.05).

Furthermore, *Mgl1* ^*−/−*^ mice had significantly lower fecal blood (FOB) scores at 12, 33, 54, 61, 68 and 75 days post‐AOM treatment than did WT mice (Figure 2e, *P < 0.05 on day 54 and ****P < 0.0001 for the other days). The area under the curve analysis confirmed that *Mgl1* ^*−/−*^ mice had lower FOB scores than WT mice (Figure 2f, **P < 0.01).

Finally, the disease activity index (DAI) score was obtained from weight, diarrhea and FOB data. The DAI of the *Mgl1* ^*−/−*^ mice was significantly lower at the end of the first (day 12) and second (day 33) DSS cycles, and at days 61, 68 and 75 compared with the WT mice (Figure 2g). A significant decrease in the AUC revealed that *Mgl1* ^*−/−*^ mice developed fewer severe hallmark signs of disease progression than WT mice (Figure 2h, **P < 0.01).

### Mgl1^−/−^ mice presented fewer and smaller tumors than the WT mice

On day 75 after CAC induction, *Mgl1* ^*−/−*^ and WT mice were euthanized to evaluate the CAC progression.

**Figure 3:**
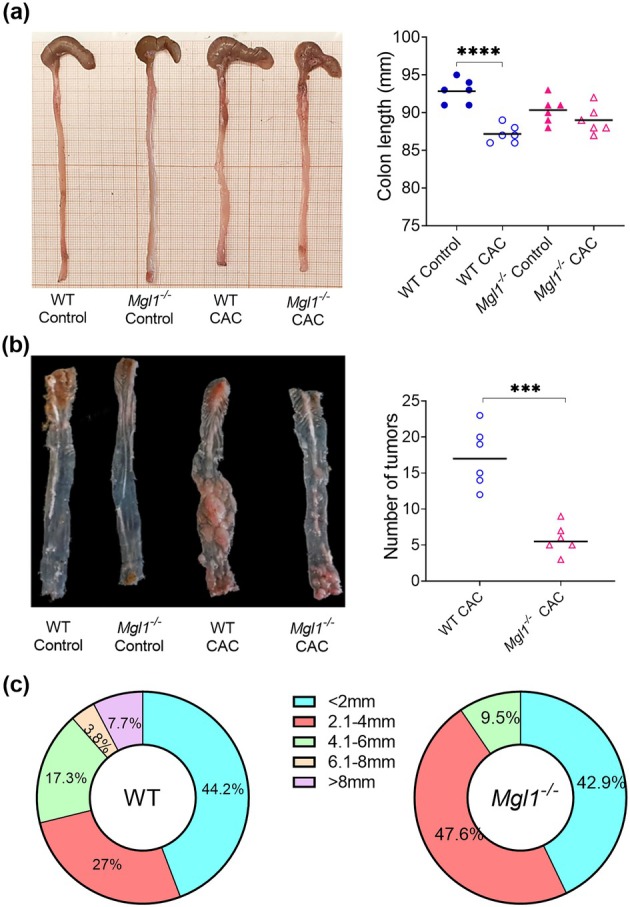
MGL1 receptor deficiency prevents colon shortening and reduces the number and size of colon tumors. Seventy‐five days after azoxymethane (AOM)‐dextran sulfate sodium (DSS) cancer induction, colons from WT control, *Mgl1* ^*−/−*^ control, WT CAC, and *Mgl1* ^*−/−*^ CAC mice were collected. **(a)** The length of the colon from the cecum to the anus was recorded. **(b)** Next, the colons were opened longitudinally to count the number of tumors and **(c)** the diameter of each tumor. The data represent three independent experiments and are presented as the mean ± standard error, *n* = 6 mice per group. The statistical significances of the differences between the *Mgl1* ^*−/−*^ CAC and WT CAC groups were analyzed using one‐way ANOVA with Tukey's multiple comparisons test and Student's paired *t*‐test as appropriate. GraphPad Prism 8.3 software was used. ***P < 0.01 and ****P < 0.0001.

Longitudinal sections of the colon were obtained for length measurement, and the intestinal lumen was exposed to obtain the number and size of the tumors. Colon shortening is a surrogate macroscopic marker of colonic injury. No significant difference in colon length was observed between WT healthy control mice (93.16 ± 0.91 mm) and *Mgl1* ^*−/−*^ healthy control mice (90.33 ± 0.71 mm). The WT CAC group exhibited significantly shorter colons than the WT control mice (Figure 3a, ****P < 0.0001). In contrast, *Mgl1* ^*−/−*^ CAC mice presented no significant differences in colon length compared with *Mgl1* ^*−/−*^ control mice (Figure 3a).

The number and size of colon tumors that develop are indicators of tumor malignancy.^23^ As expected, WT mice developed multiple tumors in the middle and distal colon at 75 days after AOM treatment. In contrast, *Mgl1* ^*−/−*^ mice developed few tumors in the distal area, with very few or no tumors in the middle area (Figure 3b). Significantly, *Mgl1* ^*−/−*^ mice developed fewer macroscopic tumors (5.83 ± 0.83) than did WT mice (17.16 ± 0.83) (Figure 3b, ***P < 0.001).

In addition, compared with those from WT mice, the colons from *Mgl1* ^*−/−*^ mice presented a decrease in the number of tumors with a mean size of 4.1–6 mm (*Mgl1* ^*−/−*^ 9.5% vs. WT 17.3%) and a more significant percentage of tumors with a mean size of 2.1–4 mm (*Mgl1* ^*−/−*^ 47.6% vs. WT 27%). Interestingly, *Mgl1* ^*−/−*^ mice did not have large tumors ≥6.1 mm, as observed in WT mice (Figure 3c). These results highlight that mMGL1 receptor deficiency may protect mice from AOM/DSS‐induced CAC initiation and progression.

### Mgl1^−/−^ mice exhibit less colon tissue damage than WT mice with colitis‐associated colorectal cancer

We assessed the impact of mMGL1 deletion on colonic tissue from healthy control and CAC mice using H&E and Alcian blue staining.

**Figure 4:**
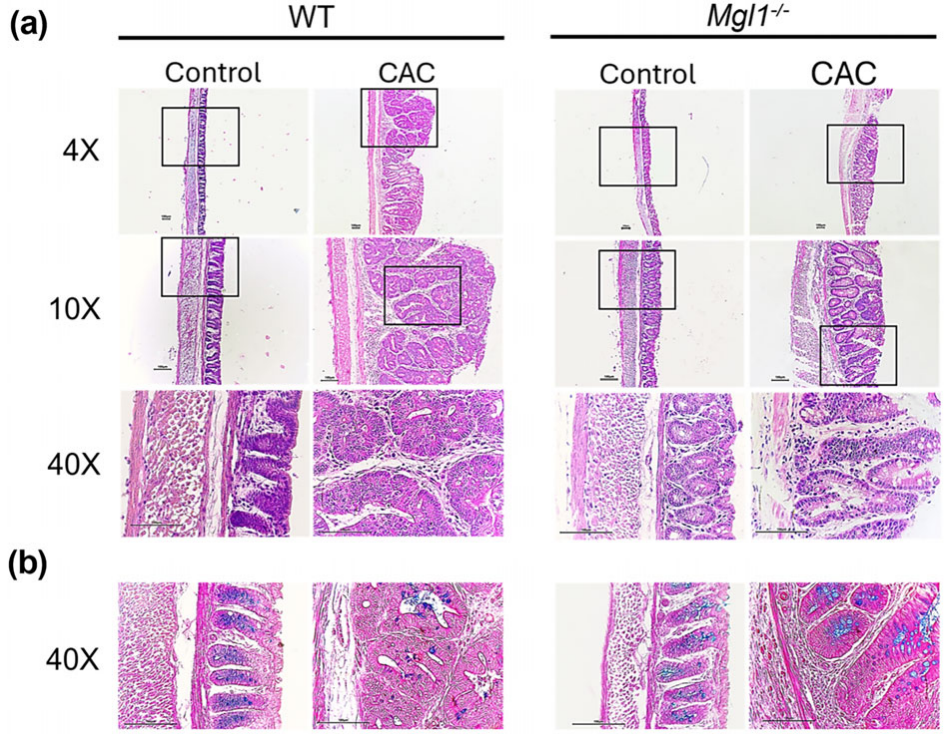
*Mgl1* ^*−/−*^ mice develop moderately differentiated colon carcinoma and maintained mucin production. Colon samples were obtained from WT CAC and *Mgl1* ^*−/−*^ CAC mice 75 days after AOM/DSS induction, and healthy mice were used as controls. **(a)** Colonic tissue morphology was examined using H&E staining. **(b)** Alcian blue staining indicates mucin‐producing goblet cells. The pictures are representative images of three independent experiments with at least 3–5 mice with CAC, and a similar number of control mice were used.

The colons of healthy control WT and *Mgl1* ^*−/−*^ mice showed typical healthy mucosa with well‐organized Lieberkühn crypts that reached the muscular layer and little cellular infiltration (Figure 4a; 4×, 10× and 40×). After CAC development, the polypoid adenocarcinomas present in the WT mice were well differentiated with atrophic crypts, lined by large columnar epithelial cells stratified with atypical hyperchromatic nuclei with the presence of nucleoli, and some glands presented a cribriform pattern. In addition, a desmoplastic pattern with abundant inflammatory infiltrate was observed. The neoplasm alternated with areas of normal mucosa that showed hyperplasia and regenerative activity with abundant inflammatory cells in the lamina propria close to the polypoid adenocarcinoma. No mucosal muscle compromise was observed (Figure 4a, 40×). In contrast, *Mgl1* ^*−/−*^ CAC mice displayed fewer and smaller polypoid adenocarcinomas, fewer aberrant crypts, less cellular infiltration and less stratified epithelium with fewer large nuclei than did WT CAC mice. Furthermore, WT CAC mice presented undifferentiated polygonal cells invading blood and lymphatic vessels, indicating increased levels of malignancy, contrary to the polypoid tumors observed in *Mgl1* ^*−/−*^ CAC mice, which did not present these histological abnormalities (Figure 4, 40×).

In addition, we compared *Mgl1* ^*−/−*^ and WT mice in terms of the production of mucin, a glycosylated protein synthesized by goblet cells that forms a physical barrier. Alcian blue staining revealed that both healthy WT and *Mgl1* ^*−/−*^ mice presented similar histological positions and numbers of normal goblet cells. WT CAC mice presented no mucin‐producing goblet cells, whereas *Mgl1* ^*−/−*^ CAC mice presented conserved crypts with mucin‐secreting goblet cells (Figure 4b). These results suggest that mMGL1 receptor deficiency partially prevents severe damage caused by AOM/DSS‐induced CAC.

### Mgl1^−/−^ mice presented increased percentages of CD4 ^+^ and CD8 ^+^ lymphocytes and decreased percentages of myeloid‐derived suppressor cells in colitis‐associated colorectal cancer

The abundant recruitment of CD8^+^ and CD4^+^ tumor specific T cells in colorectal cancer may contribute to the direct killing of malignant cells.^24^ Thus, we evaluated CD8^+^ and CD4^+^ T cell percentages at different sites after CAC‐induction. It is also known that during cancer development MDSCs (CD11b^+^Ly6G^−^Ly6C^lo^) expand and play a role in decreasing the effectiveness of anticancer therapies.^25^ MDSCs are immature immunosuppressive cells involved in tumor progression. This subgroup of cells belongs to a heterogeneous cell population that includes monocytic CD11b^+^Ly6G^−^Ly6C^hi^ (M‐MDSC) and polymorphonuclear CD11b^+^Ly6G^+^Ly6C^lo^ (PMN‐MDSCs) cells.^26^ MDSCs accumulate in the bone marrow, peripheral blood, spleen, liver, lung or tumors of various organs in most patients^27^ and mouse tumor models.^28^ In colon cancer, these cells are directly involved in tumor progression and were therefore analyzed in this study.^29^ Here, we gated FSC‐A versus CD11b^+^ cells and Ly6C versus Ly6G to identify the MDCs, M‐MDSCs and PMN‐MDSCs populations in the blood, spleen and lamina propria.

**Figure 5:**
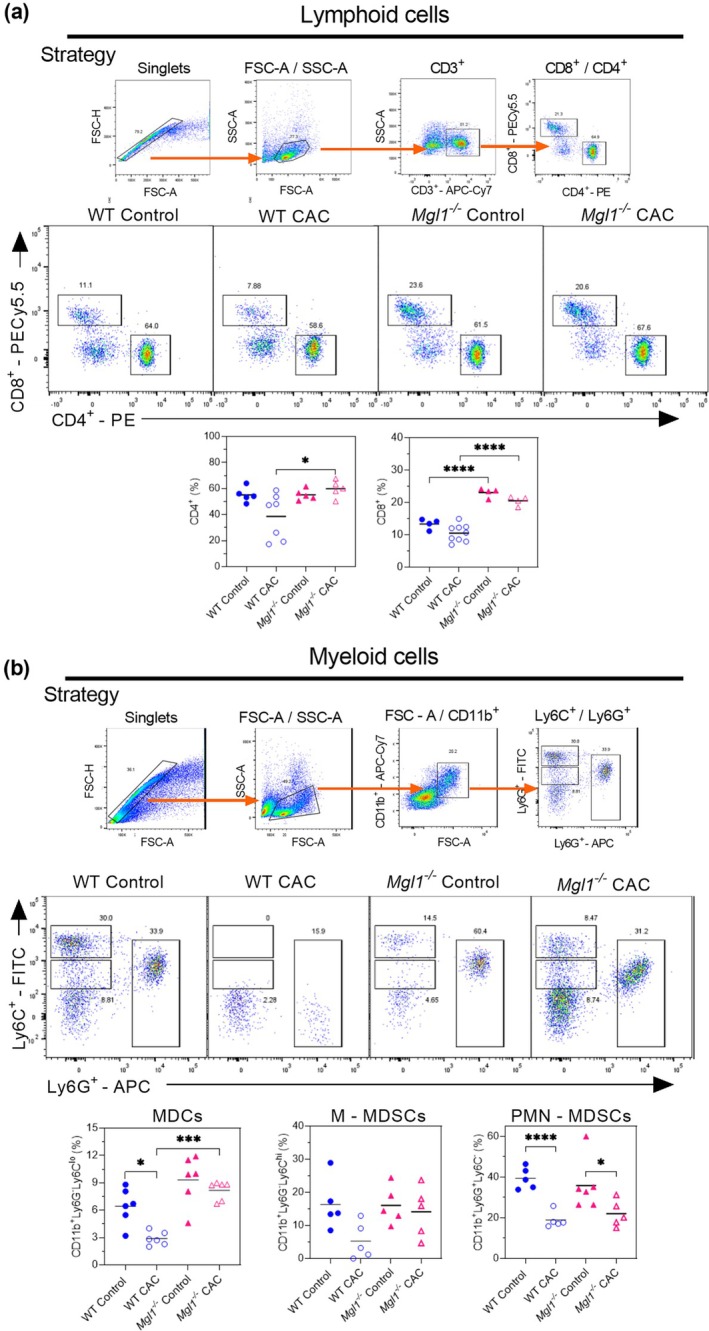
mMGL1 deficiency decreases blood PMN‐MDSCs in colitis‐associated colon cancer. **(a)** The gating strategy used to identify CD4^+^ and CD8^+^ T cells; representative dot plots of flow cytometry assays, and graphs of the percentages of CD4^+^ and CD8^+^ T cells among spleen cells. **(b)** The gating strategy. Representative dot plots of flow cytometry assays and graphs of the percentages of CD11b^+^Ly6G^−^Ly6C^lo^, CD11b^+^Ly6G^−^Ly6C^hi^ and CD11b^+^Ly6G^+^Ly6C^lo^ cells are presented. The analysis included peripheral blood cells from mice under the indicated conditions. The data represent three independent experiments and are presented as the mean ± standard error (*n* = 4–8 from one experiment). The statistical significance of differences between groups was determined using one‐way ANOVA with Tukey's multiple comparisons test. Significance is indicated as *P < 0.05, ***P < 0.001 and ****P < 0.0001 between healthy controls and CAC mice with the same genetic background and between WT CAC mice and *Mgl* ^*−/−*^ CAC mice. GraphPad Prism 8.3 software was used.

After AOM/DSS‐induced CAC, *Mgl1* ^*−/−*^ mice showed significantly greater percentages of CD4^+^ and CD8^+^ T cells in the circulation than WT CAC mice did (Figure 5a). The percentage of MDCs was also greater in *Mgl1* ^*−/−*^ CAC mice than in WT CAC mice. However, no significant differences in the percentages of M‐MDSCs or PMN‐MDSCs were detected between *Mgl1* ^−/−^ CAC and WT CAC mice (Figure 5b).

**Figure 6:**
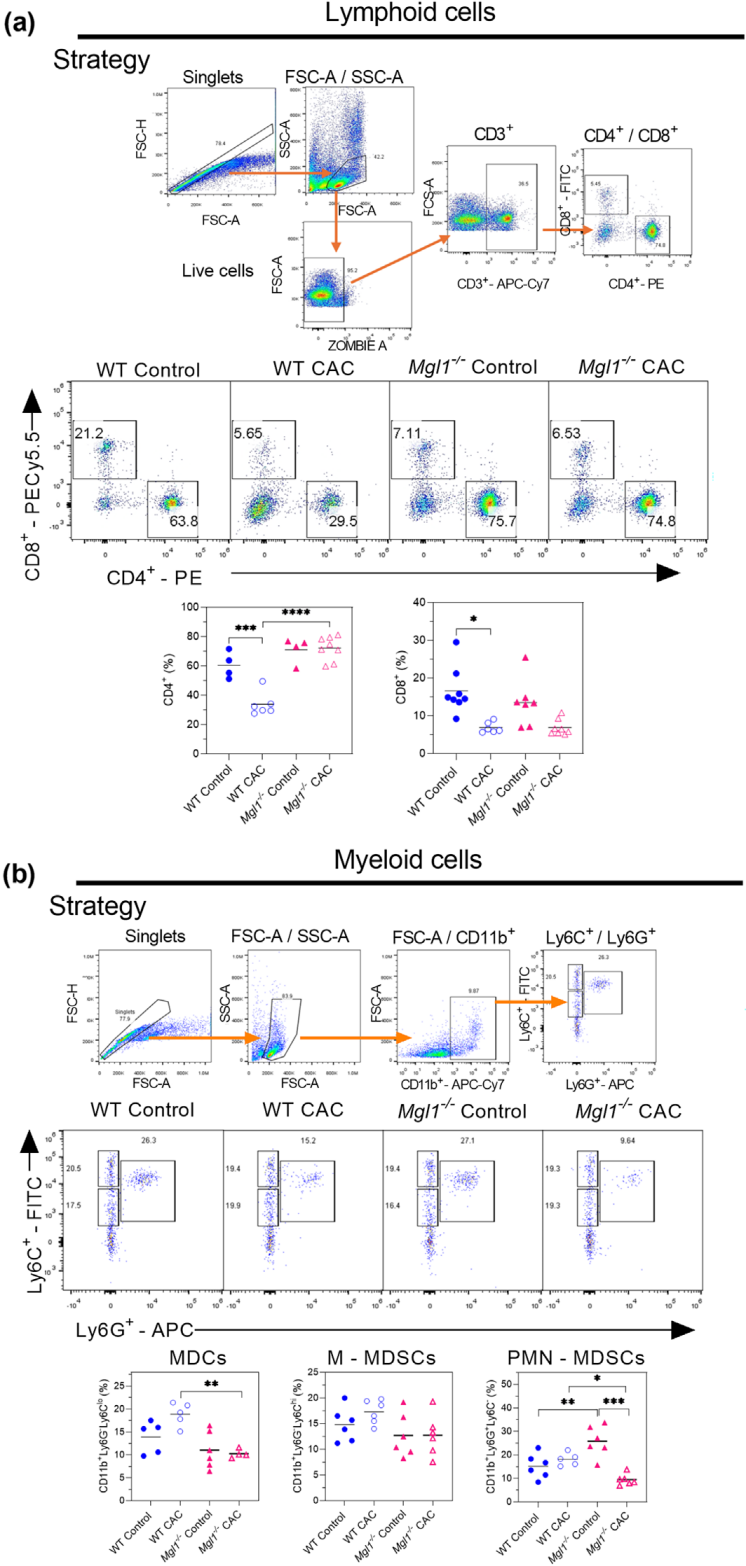
mMGL1 deficiency decreases the percentages of spleen PMN‐MDSCs in colitis‐associated colon cancer. **(a)** The gating strategy used to identify CD4^+^ and CD8^+^ T cells. Representative dot plots of flow cytometry assays and graphs of the percentages of CD4^+^ and CD8^+^ T cells among spleen cells are presented. **(b)** The gating strategy. Representative dot plots of flow cytometry assays and graphs of the percentages of CD11b^+^Ly6G^−^Ly6C^lo^, CD11b^+^Ly6G^−^Ly6C^hi^ and CD11b^+^Ly6G^+^Ly6C^lo^ cells are presented. Spleen cells from the mice were analyzed under the indicated conditions. The data represent three independent experiments and are presented as the mean ± standard error (*n* = 4–8 from one experiment). The statistical significance of differences between groups was determined using one‐way ANOVA with Tukey's multiple comparisons test. Significance is indicated as *P < 0.05, **P < 0.01, ***P < 0.001 and ****P < 0.0001 between healthy controls and CAC mice with the same genetic background and between WT CAC mice and *Mgl1* ^*−/−*^ CAC mice. GraphPad Prism 8.3 software was used.

In the spleen, *Mgl1* ^*−/−*^ CAC mice presented a significantly greater percentage of CD4^+^ T cells but a similar percentage of CD8^+^ T cells than WT CAC mice (Figure 6a). The percentage of MDCs in *Mgl1* ^*−/−*^ CAC mice was similar to that of *Mgl1* ^*−/−*^ control mice, although it was significantly lower compared with that in WT CAC mice. Nonsignificant differences were observed in the percentage of M‐MDSCs between *Mgl1* ^−/−^ and WT mice with or without CAC. Notably, after AOM/DSS‐induced CAC, the percentage of PMN‐MDSCs in *Mgl1* ^*−/−*^ mice was significantly lower than in *Mgl1* ^*−/−*^ control and WT CAC mice (Figure 6b).

**Figure 7:**
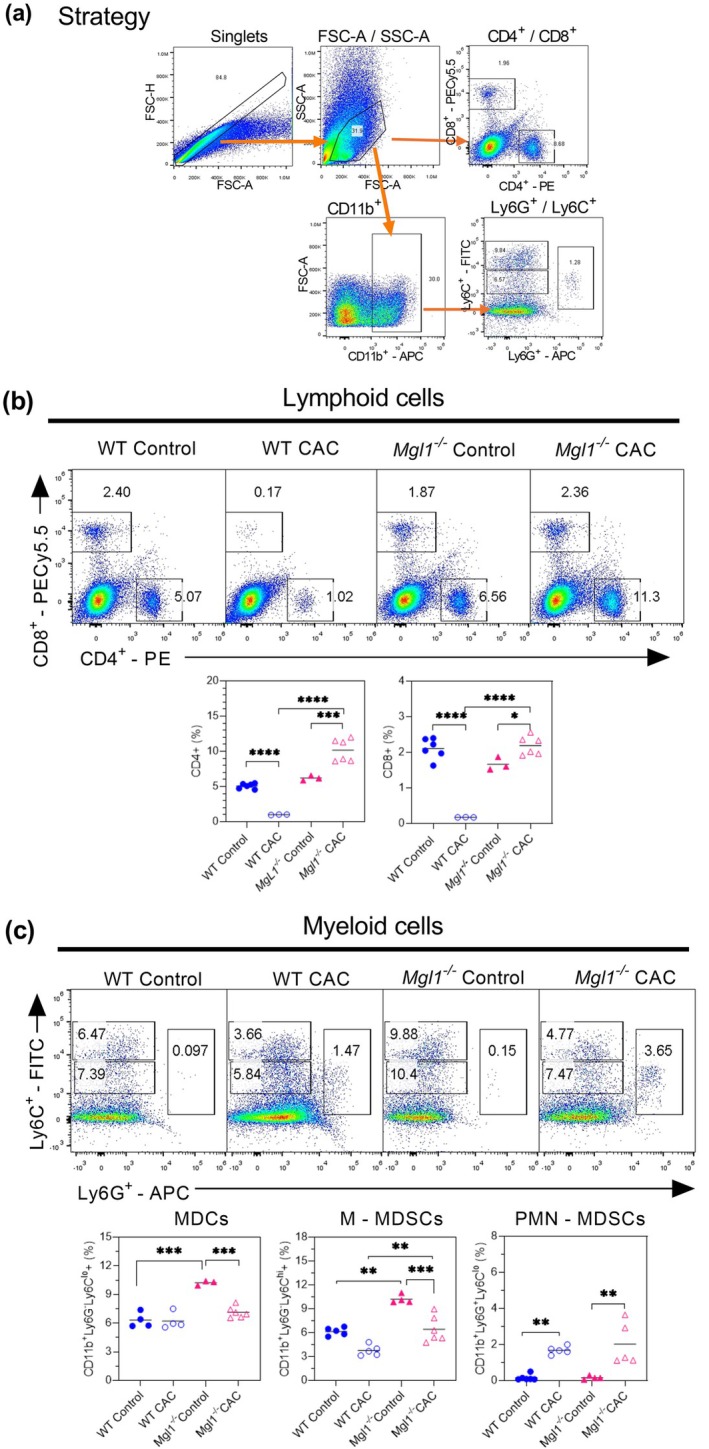
mMGL1 receptor deficiency decreases lamina propria M‐MDSCs in colitis‐associated colon cancer. **(a)** The gating strategy used to identify CD4^+^ and CD8^+^ T cells and the CD11b^+^ subpopulation of myeloid cells. **(b)** Representative dot plots and graphs of the percentages of CD4^+^ and CD8^+^ T cells among lamina propria cells. **(c)** Representative dot plots and graphs of the percentages of CD11b^+^Ly6G^−^Ly6C^lo^, CD11b^+^Ly6G^−^Ly6C^hi^ and CD11b^+^Ly6G^+^Ly6C^lo^ cells. Lamina propria cells from mice under the indicated conditions were analyzed. The data are representative of three independent experiments and are presented as the mean ± standard error (*n* = 3–6 of one experiment). The statistical significance of differences between groups was determined using one‐way ANOVA with Tukey's multiple comparisons test. Significance is indicated as *P < 0.05, **P < 0.01, ***P < 0.001 and ****P < 0.0001 between healthy controls and CAC mice with the same genetic background and between WT CAC mice and *Mgl1* ^*−/−*^ CAC mice. GraphPad Prism 8.3 software was used.

At the level of the lamina propria of tumor tissue, a significantly greater percentage of CD4^+^ and CD8^+^ T cells was detected in *Mgl1* ^*−/−*^ CAC mice than in WT CAC mice (Figure 7a). The percentages of MDCs and M–MDSCs were significantly lower in *Mgl1* ^*−/−*^ CAC mice than in *Mgl1* ^*−/−*^ control mice. Moreover, the percentages of PMN‐MDSCs were significantly greater in WT CAC and *Mgl1* ^−/−^ CAC mice than in their respective controls (Figure 7b).

Taken together, these data suggest that mMGL1 receptor may alter CD4^+^ and CD8^+^ T‐cell functions, mainly in the tumor microenvironment, potentially contributing to tumor growth. Furthermore, mMGL1 receptor deletion led to a reduction of the M‐MDSCs percentage in the colon tumor tissue.

### Mgl1^−/−^ mice displayed decreased recruitment of macrophages and NKs, lower iNOS, and arginase expression in the tumor microenvironment in colitis‐associated colorectal cancer

**Figure 8:**
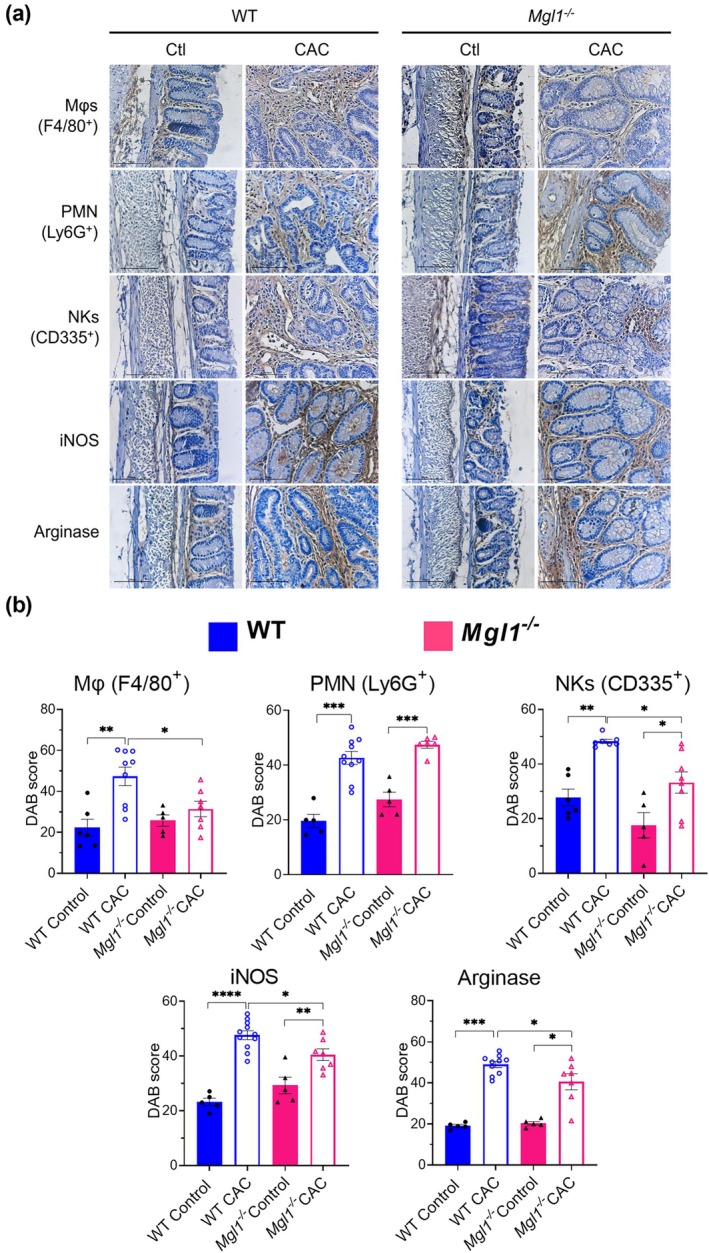
mMGL1 deficiency decreases the abundance of Mφs, NK cells, iNOS and arginase in colon tumor infiltration. The presence of Mφs (F4/80^+^), PMN (Ly6G^+^) and NK cells (CD335^+^), and iNOS and arginase in the colons of *Mgl1* ^*−/−*^ and WT mice was assessed using immunohistochemistry. **(a)** Representative immunostaining images of the descending colon captured at 40× magnification. **(b)** The total positive percentage contribution of the DAB color spectra was measured as the presence/absence of F4/80^+^, Ly6G^+^, CD335^+^, iNOS and arginase in the colon sections. The total percentage of positive cells was calculated using the IHC profiler plugin for Fiji software from ImageJ. The DAB score was calculated as the percentage of total positive pixels in the DAB color spectra. The pictures are representative images of three independent experiments with at least 3–5 mice per group, and the data are presented as the mean ± standard error; *P < 0.05, **P < 0.01, ***P < 0.001 and ****P < 0.0001 was calculated by one‐way ANOVA with Tukey's multiple comparisons test. GraphPad Prism 8.3 software was used.

To characterize the immune cell populations in the colon environment, we used immunohistochemical assays to examine the presence of Mφs (F4/80^+^), PMN (Ly6G^+^) and NK cells (CD335^+^), and iNOS and arginase in colon tissue from WT and *Mgl1* ^*−/−*^ mice with and without CAC (Figure 8a; Supplementary figure 1).

Immunohistochemical analysis revealed normal cellular infiltration in the colonic tissue in healthy control WT and *Mgl1* ^*−/−*^ mice. After AOM/DSS‐induced CAC, the polypoid tumors of the WT mice presented abundant inflammatory infiltrates characterized by the presence of Mφs, PMNs, NKs, iNOS and arginase levels on the lax tissue of the lamina propria in both normal and neoplastic tissues (Figure 8a). Remarkably, compared with WT CAC mice, *Mgl1* ^*−/−*^ CAC mice presented less infiltration of Mφs, NKs and arginase in the lax tissue of the lamina propria. No significant differences in PMN infiltration were detected between *Mgl1* ^*−/−*^ CAC mice and the WT CAC mice (Figure 8b).

These results suggest that the interaction of the mMGL1 receptor with aberrantly glycosylated proteins may trigger the recruitment of inflammatory and immunosuppressive cells and increase iNOS production in the tumor microenvironment.

## DISCUSSION

The interaction between tumor‐associated antigens and antigen‐presenting cells such as Mφs and DCs is critical for the specific generated immune response. Several studies indicate a close association between aberrant protein glycosylation and cancer progression and metastasis.^30^ Epithelial MUC1 is a high‐molecular‐weight membrane glycoprotein that is frequently overexpressed and aberrantly glycosylated in adenocarcinoma. Certain glycan epitopes of MUC1, such as the Tn‐antigen, TF‐antigen and their sialylated forms, are exposed during malignant transformation,^31^ and MGL receptor was shown to play a role as a recognition molecule on Mφs for tumor cells.^32^

*In vitro* studies using colon tumor human cell lines have indicated that MGL is the receptor that recognizes and binds to MUC1–Tn antigen;^33^ however, the role of the interaction between the MUC1‐Tn antigen and antigen‐presenting cells in the biology of CAC tumors is unknown. Thus, the present study was performed to investigate *in vivo* the relevance of the mMGL1 receptor in the development of CAC using *Mgl1* ^*−/−*^ and WT mice.

To validate our findings, we first showed that both Mφs and DCs from *Mgl1* ^*−/−*^ mice presented no detectable mMGL1 expression, whereas WT mice presented increased mMGL1 expression after LPS stimulation. Furthermore, mMGL2 expression remained intact, and its expression was similar in Mφs and DCs from WT and *Mgl1* ^*−/−*^ mice as reported previously.^34^ In addition, we detected no expression of MGL1 or MGL2 in naïve B lymphocytes, or in CD4^+^ and CD8^+^ T cells stimulated with concanavalin (Con)A or LPS (Supplementary figure 2). In line with this, Tsuiji *et al*. investigated the distribution of mRNA for *Mgl1* and *Mgl2* in 10 mouse cell lines, including L929 (fibroblast line), JLS‐V9 (bone marrow‐derived fibroblast‐like cell line), EL4 (thymoma cell line), RL 1 (lymphoma cell line), YAC‐1 (lymphoma cell line), BCL1‐B20 (malignant B cells), P815 (mastocytoma cells), P388 (Mφ‐like lymphoid cell line), M1 (myeloblastic leukemia cells) and RAW264.7 (Mφ‐like cell line). They reported that *mMgl1* and *mMgl2* were expressed only in the Mφ‐like cell lines, P388 and RAW264.7.^12^ Despite these reports and the limitations of our study, relevant cells in the context of CAC, such as exhausted T cells, neutrophils, NK cells and other cells that constitute the tumor tissue microenvironment should be studied to affirm, or rule out, that MGL1/2 expression is restricted to Mφs, DCs and mast cells, as has been suggested previously, and to confirm whether the MGL1 receptor is critical for the CAC outcome.

Moreover, we demonstrated that the tumor tissue from the AOM/DSS‐induced CAC mouse model contained strongly glycosylated protein patterns with molecular weights greater than 60 kDa, which aligns with previous *in vitro* assays using human colon cancer cell lines.^17^ These results demonstrate that colon tumor tissue displays glycosylated antigens potentially recognized by the mMGL1 receptor; here, we assessed whether these interactions favor or not CAC tumor progression in a mouse model.

Our results suggest for the first time *in vivo* that the mMGL1 receptor may play a protumorigenic role in the development of CAC, given that mMGL1 deletion significantly reduces the signs of CAC by maintaining stable body weight and decreasing the incidence of diarrhea and fecal occult blood. Additionally, *Mgl1* ^*−/−*^ mice developed fewer and smaller tumors (less than 5 mm) than WT mice, suggesting that the presence of mMGL1 receptor may trigger carcinogenesis by altering the tumor microenvironment.

Interactions between the hMGL receptor and tumor‐associated Tn antigens have been reported in colon carcinoma^31^ and recently reported in a transgenic model of breast cancer, but its link to immune evasion or immunosuppression was not evaluated.^35^ Thus, the immunological mechanisms involved in this interaction have not been described. Therefore, we evaluated the percentages of CD4^+^ and CD8^+^ T cells, which are among the main effector cells involved in the eradication of tumor, as well as MDSCs, which can interact with other immune cells to inhibit the immune response and ensure tumor cell survival.^36^ We found that mMGL1 deletion contributed to the increased percentage of CD4^+^ and CD8^+^ T cells in the circulation and lamina propria while downregulating the MDCs and M‐MDSCs recruitment in the lamina propria of colon tumors. This increase in CD4^+^ and CD8^+^ cells may be related to the reported ability of MGL receptor to bind to CD45 on effector T cells, reducing its proliferation and favoring apoptosis.^37^

The absence of mMGL1 receptor also appears to contribute to reducing the expansion of M‐MDSC in the tumor microenvironment; consequently, the percentages of CD4^+^ and CD8^+^ T cells increased, which may be associated with the lower number and size of colon tumors in *Mgl1* ^*−/−*^ CAC mice. In this context, the persistent inflammation that occurs in CAC may disrupt myelopoiesis, leading to the generation of immature myeloid cells, such as MDSCs.^38^ These cells are associated with a reduction in the adaptative immune response to tumor MUC1 by lowering the production of specific antibodies and reducing the activation and proliferation of tumor‐specific T cells while stimulating Treg development.^29^

The suppressive role of MDSCs in cancer is associated with the activation of inducible nitric oxide synthase (iNOS) and arginase‐1. These enzymes are responsible for the metabolism of L‐arginine, which is essential for T cell proliferation and function. The nitric oxide and reactive oxygen species produced by MDSCs are involved in the inactivation of the T‐cell receptor (TCR), causing a decrease in the expression of the CD3ζ chain and inducing T‐cell apoptosis.^39^ Thus, low recruitment of MDSCs in the absence of mMGL1 may help to reduce colon tumorigenesis.

In addition to immune evasion, tumor‐promoting inflammation, characterized by the presence of M‐MDSCs and critical inflammatory mediators such as monocytes, Mφs, neutrophils and NK cells, is a hallmark of CAC.^39^ Here, we demonstrated that colon tissue from *Mgl1* ^*−/−*^ CAC mice exhibited less infiltration of Mφs and NK cells, while lowering iNOS and arginase expression levels, than in WT CAC mice. These findings align with those recently reported in a transgenic breast cancer model where high MGL binding to aberrantly glycosylated tissue increased the recruitment of DCs and Mφs at the tumor.^35^ In addition, we observed a reduction in IL‐17A and IL‐4 levels but increased IFN‐γ RNAm levels in the colonic tumors of the *Mgl1* ^*−/−*^ mice (Supplementary figure 3); IL‐17A and IL‐4 are cytokines supporting colon tumor growth, whereas IFN‐γ has antitumorigenic functions.^24^ This observation suggests that the mMGL1 receptor promotes inflammation.

Overactivation of the extracellular signal‐regulated kinase (ERK) 1/2 signaling pathway has been recognized as an inducer of colorectal cancer by promoting inflammation, cell proliferation, angiogenesis and metastasis.^40^ Consistent with these findings, we observed that colonic ERK1/2 signaling was significantly reduced in *Mgl1* ^*−/−*^ CAC mice compared with WT CAC mice (Supplementary figure 4). These results suggest that recognition of aberrantly glycosylated antigens in tumor cells by the mMGL1 receptor could activate the intracellular signaling of MAPKs, such as ERK1/2, thereby promoting inflammation and MDSCs differentiation. However, future experiments are needed to test this hypothesis.

In conclusion, these data demonstrate for the first time *in vivo* that the absence of the mMGL1 receptor restrains MDSC expansion; reduces the infiltration of inflammatory mediators such as Mφs, NKs, iNOS and arginase 1; and increases the percentage of CD4^+^ and CD8^+^ T cells in colon tumors, thereby reducing tumor growth in the colonic mucosa. Thus, recognition of glycosylated proteins by the mMGL1 receptor exacerbates CAC in mice through upregulation of the proinflammatory microenvironment. These observations suggest that the MGL receptor may be a therapeutic target and provide guidelines for future studies investigating the role of the MGL receptor in human colorectal cancer.

## METHODS

### Mice

Nine‐week‐old *Mgl1* ^−/−^ mice on a C57BL/6 genetic background were backcrossed for over 10 generations.^41^ The *Mgl1* ^−/−^ and WT mice were maintained in a pathogen‐free environment at the Facultad de Estudios Superiores (FES)‐Iztacala animal facility. Mgl1^*−/−*^ mouse genotyping is routinely performed on DNA isolated from tail snips via polymerase chain reaction (PCR).^42^ PCR was performed using the primers indicated in Supplementary table 1 (all synthesized by Sigma–Aldrich, Mexico City, Mexico). PCR for the amplification of MGL and NEO was performed with Taq DNA polymerase (Ampliqon, Bioreagents, and Molecular Diagnostics, Odense, Denmark) following the manufacturer's instructions. PCR products of 714 bp, corresponding to MGL, and 492 bp, corresponding to NEO, were visualized to identify the WT and *Mgl1* ^*−/−*^ genotypes, respectively (Supplementary figure 5). The PCR products were analyzed using electrophoresis on a 1.5% agarose gel and viewed under UV light (Bio‐Rad, Hercules, CA, USA).

### Ethics statement

All experimental procedures and the number of mice used were intended to reduce suffering. The studies were conducted in accordance with the ethical standards approved and performed under strict accordance with the Guidelines for the Care and Use of Laboratory Animals adopted by the US National Institutes of Health and the Mexican Guideline Regulation of Animal Care and Maintenance (NOM–062–ZOO‐1999, 2002), and the Faculty Internal Ethics Committee approved the experimental protocol (Protocol CE/FACI/082020/1310).

### AOM‐DSS‐induced CAC mouse model

A slightly modified rodent colon carcinogenesis protocol was used.^43^ Briefly, the mice received an intraperitoneal injection of 10 mg kg^−1^ azoxymethane (AOM; Sigma–Aldrich). Five days later, 2% dextran sulfate sodium (DSS, MW: 40000, Alfa Aesar, Ward Hill, MA, USA) was administered in the drinking water *ad libitum* for 7 days. The mice were then provided tap water for 14 days and subjected to two additional cycles of 1.5% DSS. All the mice were euthanized on day 75, and the colons were removed, weighed and subjected to macroscopic inspection and histopathological examination. Tumors were measured with a digital caliper, and the tumor size was determined using the following formula: tumor size mm^3^ = length × width^2^/2.^23^ The tumor burden per mouse is the sum of all tumor sizes.

### Lectin blot

Tumors were cryofractured, and protein was extracted using RIPA lysis buffer (20 mM Tris HCl 8.0, 300 mM NaCl, 2% (v/v) NP – 40, 1% (w/v) sodium deoxycholate, 0.2% SDS, 2 mM mercaptoethanol, 20 mM NaF and 2 mM Na_3_VO_4_, all from Sigma–Aldrich, with protease and phosphatase inhibitors from Roche Diagnostic, Basel, Switzerland). Protein samples (10 μg) were separated on a gradient of 4–20% SDS–PAGE gel and transferred to a PVDF (polyvinylidene difluoride) membrane (0.22 M, Millipore, Bedford, MA, USA) using a western blotting unit (Bio‐Rad). The membrane was blocked overnight at 4°C with 5% (w/v) bovine serum albumin in PBS (pH 7.2), washed thoroughly with 0.05% PBS/Tween, and incubated with horseradish peroxidase (HRP)‐conjugated lectin from *Triticum vulgaris* (wheat germ agglutinin lectin (WGA), Sigma–Aldrich). The signal was revealed using SuperSignal West Femto (Thermo Fisher Scientific, Waltham, MA, USA) and then digitalized and analyzed using a UVITEC chemiluminescence imaging system (Cambridge, UK) with Alliance software. The relative density of the protein bands was analyzed using the Image Lab software version 6.1.0 build 7 standard editions from Bio‐Rad Laboratories, Inc. and was divided into four molecular weight (MW) categories 260–100, 100–45, 45–25, 25–3 kilodaltons (kDa).

### Disease progression evaluation

The clinical symptoms in each experimental group, body weight, stool consistency and blood in the stool were recorded weekly using the modified disease activity index (DAI) grading system, which was calculated by assigning established scores. Diarrhea was scored as follows: 0, normal; 2, loose stools; and 4, watery diarrhea. Blood in the stool was scored as follows: 0, normal; 2, slight bleeding; and 4, gross bleeding. Weight loss was scored as follows: 0, none; 1, 1–5%; 2, 5–10%; 3, 10–15% and 4, > 15%. The DAI is the sum of diarrhea, blood in the stool, and weight loss scores divided by three.^44^

### Cell preparation and culture conditions

Bone marrow‐derived DCs and Mφs from *Mgl1* ^*−/−*^ and WT mice were obtained and differentiated according to a previously described protocol.^45^ Briefly, bone marrow cells were flushed from the hind limb bones (femora and tibiae) with DMEM supplemented with 10% fetal calf serum (FCS), 2 mM L‐glutamine, 0.25 U mL^−1^ penicillin and 100 μg m L^−1^ streptomycin (all from Gibco, Thermo Fisher Scientific). Red blood cells were lysed with 0.83% ammonium chloride (Sigma–Aldrich), after 72 h of incubation with 20 ng mL^−1^ recombinant murine granulocyte‐macrophage colony‐stimulating factor (rGM–CSF; Peprotech, Mexico City, Mexico) in complete DMEM. The nonadherent cells were discarded, and the remaining cells were cultured in fresh DMEM + rGM–CSF for 6 days. Mature cells were stained with fluorochrome‐conjugated antibodies and subjected to flow cytometry in the following combinations: nonadherent CD11b^+^CD11c^+^ cells (70–80%, DC) and adherent CD11b^+^CD11C^−^ cells (70–80% Mφs). Blood and spleen cells were processed for flow cytometry analysis.

### Flow cytometry analysis

Bone marrow‐derived murine DCs and Mφs were transferred to 24‐well culture plates (Costar, Cambridge, MA, USA) at 5 × 10^5^ cells/well and stimulated with LPS (100 ng mL^−1^) for 24 h. Afterward, the cells were harvested at a density of 1 × 10^6^ cells mL^−1^ in DMEM. The cells were incubated with the following fluorochrome‐conjugated antibodies: PerCP/Cy5.5‐conjugated anti‐CD11b, FITC‐conjugated anti‐CD11c, PE‐conjugated anti‐MGL1 and APC‐conjugated anti‐MGL2 (all from Biolegend, San Diego, CA, USA).

The colonic lamina propria cells were isolated using a lamina propria dissociation kit (Miltenyi Biotec, Bergisch Gladbach, Germany) as indicated by the supplier. Briefly, colon sections were flushed with ice‐cold PBS, cut longitudinally, and then laterally into pieces approximately 0.5 cm in length. Lamina propria cells were isolated, performing three pre‐digestion incubations in HBSS containing 2 mM EDTA, 2% FBS and 1x penicillin–streptomycin‐glutamine cocktail (Gibco) at 37°C. This was followed by passage through 100 μm Falcon cell strainers (Becton Dickinson, Sunnyvale, CA) to remove epithelial cells and a digestion step with the enzyme mixture containing 100 μL of enzyme D, 50 μL of enzyme R, and 12.5 μL of enzyme A. After digestion, the cell suspension was filtered through a 100‐μm Falcon cell strainer (Becton) and centrifuged at 300 × *g* for 10 min to pellet the lamina propria cells, which were then resuspended and collected for FACS analysis.

Single‐cell suspensions of the colonic lamina propria spleen and blood cells from *Mgl1* ^*−/−*^ and WT mice were subjected to flow cytometry using the following myeloid‐derived suppressor cell (MDSC) antibodies: APCy7‐conjugated anti‐CD11b, APC‐conjugated anti‐Ly6G and FITC‐conjugated anti‐Ly6C (all from BioLegend). In addition, spleen cells were incubated with the following lymphocyte antibodies: PerCP/Cy5.5‐conjugated anti‐CD3, APC‐conjugated anti‐CD4 and FITC‐conjugated anti‐CD8. In all cases, the cells were washed three times with FACS buffer, fixed in 0.8% paraformaldehyde, and acquired (1 × 10^5^ events) (Attune NxT, Thermo Fisher Scientific). FlowJo v10 software was used for analysis.

### Histology and immunohistochemistry

Longitudinal colon sections from *Mgl1* ^*−/−*^ and WT mice were obtained and fixed by immersion in 4% buffered paraformaldehyde, dehydrated with increasing concentrations of ethanol, embedded in paraffin and cut into 5‐μm sections. The sections were stained with hematoxylin and eosin (H&E; for pathologic evaluation) or Alcian blue and contrasted with H&E (to quantify goblet cells). A histopathologist (in a blind way) evaluated all the samples under a light microscope (UNICO, Princeton, NJ, USA).

As reported previously, paraffin‐embedded colon sections were also subjected to immunohistochemistry (IHC) assays.^46^ The sections were incubated overnight at 4°C with the following primary antibodies: anti‐F4/80, anti‐Ly6G, anti‐iNOS (BioLegend, San Diego, CA, USA) and anti‐CD335 and anti‐arginase 1 (BD Biosciences, NJ, USA). Then, the sections were developed following the conventional technique. All immunostained slide images were captured at 10× and 40× objective lenses using a Unico G380 microscope (Unico) equipped with an AmScope camera (AmScope, United States). The numbers of Mφs (F4/80^+^), PMN (Ly6G^+^) and NK cells (CD335^+^), and iNOS and arginase (DAB‐HRP reaction) were measured semiquantitatively in the images captured using the 10× objective with the “FIJI” version of ImageJ software using the plugin IHC profiler.

### Statistical analysis

The differences between groups were evaluated using one‐way ANOVA, two‐way ANOVA, or a Student's unpaired *t*‐test with Welch's correction, as appropriate. *P < 0.05; **P < 0.01; ***P < 0.001; ****P < 0.0001 indicate statistical significance. Analyses were performed using Graph Pad Prism 8.3 software (Graph Pad Software, Inc., San Diego, CA, USA).

## AUTHOR CONTRIBUTIONS

**Oscar Nieto‐Yañez:** Methodology; writing – original draft. **Sonia H Navia:** Data curation; formal analysis; methodology; writing – review and editing. **Imelda Juárez‐Avelar:** Data curation; methodology; project administration. **Tonathiu Rodríguez:** Formal analysis; methodology; writing – original draft. **Antonio Andrade‐Meza:** Formal analysis. **Betsaida J Ortiz‐Sánchez:** Data curation; methodology; writing – review and editing. **Mónica G Mendoza‐Rodríguez:** Formal analysis; methodology; writing – review and editing. **Jonadab E Olguín:** Data curation; formal analysis; methodology. **José L Reyes:** Writing – review and editing. **Daniel Montes de Oca‐Samperio:** Methodology. **Citlaltepetl Salinas Lara:** Formal analysis. **Luis I Terrazas:** Conceptualization; data curation; formal analysis; writing – review and editing. **Miriam Rodriguez‐Sosa:** Conceptualization; data curation; formal analysis; funding acquisition; investigation; methodology; project administration; resources; supervision; validation; visualization; writing – review and editing.

## CONFLICT OF INTEREST

The authors declare no conflicts of interest.

## Supporting information


Supplementary figure 1.
**Supplementary figure 2**.
**Supplementary figure 3**.
**Supplementary figure 4**.
**Supplementary figure 5**.
**Supplementary table 1**.

## Data Availability

The data that support the findings of this study are available from the corresponding author upon reasonable request.

## References

[1] Mattiuzzi C , Sanchis‐Gomar F , Lippi G . Concise update on colorectal cancer epidemiology. Ann Transl Med 2019; 7: 609. 32047770 10.21037/atm.2019.07.91 PMC7011596

[2] Bray F , Laversanne M , Sung H , *et al*. Global cancer statistics 2022: GLOBOCAN estimates of incidence and mortality worldwide for 36 cancers in 185 countries. CA Cancer J Clin 2024; 74: 229–263. 38572751 10.3322/caac.21834

[3] Andrade‐Meza A , Arias‐Romero LE , Armas‐López L , *et al*. Mexican colorectal cancer research consortium (MEX‐CCRC): etiology, diagnosis/prognosis, and innovative therapies. Int J Mol Sci 2023; 24: 2115. 36768437 10.3390/ijms24032115 PMC9917340

[4] Azizian‐Farsani F , Abedpoor N , Hasan Sheikhha M , Gure AO , Nasr‐Esfahani MH , Ghaedi K . Receptor for advanced glycation end products acts as a fuel to colorectal cancer development. Front Oncol 2020; 10: 552283. 33117687 10.3389/fonc.2020.552283 PMC7551201

[5] Chaib M , Chauhan SC , Makowski L . Friend or foe? Recent strategies to target myeloid cells in cancer. Front Cell Dev Biol 2020; 8: 351. 32509781 10.3389/fcell.2020.00351 PMC7249856

[6] Kovács T , Mikó E , Ujlaki G , Sári Z , Bai P . The microbiome as a component of the tumor microenvironment. Adv Exp Med Biol 2020; 1225: 137–153. 32030653 10.1007/978-3-030-35727-6_10

[7] Brown GD , Willment JA , Whitehead L . C‐type lectins in immunity and homeostasis. Nat Rev Immunol 2018; 18: 374–389. 29581532 10.1038/s41577-018-0004-8

[8] Vázquez‐Mendoza A , Carrero JC , Rodriguez‐Sosa M . Parasitic infections: a role for C‐type lectins receptors. Biomed Res Int 2013; 2013: 456352. 23509724 10.1155/2013/456352 PMC3581113

[9] Yan H , Kamiya T , Suabjakyong P , Tsuji NM . Targeting C‐type lectin receptors for cancer immunity. Front Immunol 2015; 6: 408. 26379663 10.3389/fimmu.2015.00408 PMC4547497

[10] Ding D , Yao Y , Zhang S , Su C , Zhang Y . C‐type lectins facilitate tumor metastasis. Oncol Lett 2017; 13: 13–21. 28123516 10.3892/ol.2016.5431 PMC5245148

[11] Vukman KV , Ravida A , Aldridge AM , O'Neill SM . Mannose receptor and macrophage galactose‐type lectin are involved in *Bordetella pertussis* mast cell interaction. J Leukoc Biol 2013; 94: 439–448. 23794711 10.1189/jlb.0313130

[12] Tsuiji M , Fujimori M , Ohashi Y , *et al*. Molecular cloning and characterization of a novel mouse macrophage C‐type lectin, mMGL2, which has a distinct carbohydrate specificity from mMGL1. J Biol Chem 2002; 277: 901. 10.1074/jbc.M203774200 12016228

[13] Zizzari IG , Napoletano C , Battisti F , *et al*. MGL receptor and immunity: when the ligand can make the difference. J Immunol Res 2015; 2015: 450695. 26839900 10.1155/2015/450695 PMC4709716

[14] Drickamer K . C‐type lectin‐like domains. Curr Opin Struct Biol 1999; 9: 585–590. 10508765 10.1016/s0959-440x(99)00009-3

[15] Singh SK , Streng‐Ouwehand I , Litjens M , *et al*. Characterization of murine MGL1 and MGL2 C‐type lectins: distinct glycan specificities and tumor binding properties. Mol Immunol 2009; 46: 1240–1249. 19162326 10.1016/j.molimm.2008.11.021

[16] Suzuki N , Yamamoto K , Toyoshima S , Osawa T , Irimura T . Molecular cloning and expression of cDNA encoding human macrophage C‐type lectin. Its unique carbohydrate binding specificity for Tn antigen. J Immunol 1996; 156: 128–135. 8598452

[17] Pirro M , Mohammed Y , van Vliet SJ , *et al*. N‐glycoproteins have a major role in MGL binding to colorectal cancer cell lines: Associations with overall proteome diversity. Int J Mol Sci 2020; 21: 5522. 32752259 10.3390/ijms21155522 PMC7432225

[18] Ju T , Otto VI , Cummings RD . The Tn antigen‐structural simplicity and biological complexity. Angew Chem Int Ed Engl 2011; 50: 1770–1791. 21259410 10.1002/anie.201002313 PMC7159538

[19] Pirro M , Rombouts Y , Stella A , *et al*. Characterization of macrophage galactose‐type lectin (MGL) ligands in colorectal cancer cell lines. Biochim Biophys Acta Gen Subj 2020; 1864: 129513. 31911241 10.1016/j.bbagen.2020.129513

[20] Lenos K , Goos JA , Vuist IM , *et al*. MGL ligand expression is correlated to BRAF mutation and associated with poor survival of stage III colon cancer patients. Oncotarget 2015; 6: 290. 10.18632/oncotarget.4495 PMC4694901 26172302

[21] Iskratsch T , Braun A , Paschinger K , Wilson IB . Specificity analysis of lectins and antibodies using remodeled glycoproteins. Anal Biochem 2009; 386: 133–146. 19123999 10.1016/j.ab.2008.12.005

[22] Malagolini N , Santini D , Chiricolo M , Dall'Olio F . Biosynthesis and expression of the Sda and sialyl Lewis × antigens in normal and cancer colon. Glycobiology 2007; 17: 688–697. 17395692 10.1093/glycob/cwm040

[23] Pacheco‐Fernandez T , Juarez‐Avelar I , Illescas O , *et al*. Macrophage migration inhibitory factor promotes the interaction between the tumor, macrophages, and T cells to regulate the progression of chemically induced colitis‐associated colorectal cancer. Mediators Inflamm 2019; 2019: 2056085. 31360118 10.1155/2019/2056085 PMC6652048

[24] Zheng Z , Wieder T , Mauerer B , Schafer L , Kesselring R , Braumuller H . T cells in colorectal cancer: unravelling the function of different T cell subsets in the tumor microenvironment. Int J Mol Sci 2023; 24: 11673. 37511431 10.3390/ijms241411673 PMC10380781

[25] Wu SY , Chiang CS . Distinct role of CD11b(+)Ly6G(−)Ly6C(−) myeloid‐derived cells on the progression of the primary tumor and therapy‐associated recurrent brain tumor. Cells 2019; 9: 51. 31878276 10.3390/cells9010051 PMC7016541

[26] Zhao Y , Wu T , Shao S , Shi B , Zhao Y . Phenotype, development, and biological function of myeloid‐derived suppressor cells. Onco Targets Ther 2016; 5: e1004983. 10.1080/2162402X.2015.1004983 PMC4801459 27057424

[27] Tam JW , Kullas AL , Mena P , Bliska JB , van der Velden AW . CD11b^+^ Ly6C^hi^ Ly6G^‐^ immature myeloid cells recruited in response to Salmonella enterica Serovar Typhimurium infection exhibit protective and immunosuppressive properties. Infect Immun 2014; 82: 2606–2614. 24711563 10.1128/IAI.01590-13 PMC4019163

[28] Zhang QQ , Hu XW , Liu YL , *et al*. CD11b deficiency suppresses intestinal tumor growth by reducing myeloid cell recruitment. Sci Rep 2015; 5: 15948. 26526388 10.1038/srep15948 PMC4630647

[29] Sieminska I , Baran J . Myeloid‐derived suppressor cells in colorectal cancer. Front Immunol 2020; 11: 1526. 32849517 10.3389/fimmu.2020.01526 PMC7426395

[30] Lin Y , Lubman DM . The role of N‐glycosylation in cancer. Acta Pharm Sin B 2024; 14: 1098–1110. 38486989 10.1016/j.apsb.2023.10.014 PMC10935144

[31] Saeland E , van Vliet SJ , Backstrom M , *et al*. The C‐type lectin MGL expressed by dendritic cells detects glycan changes on MUC1 in colon carcinoma. Cancer Immunol Immunother 2007; 56: 1225–1236. 17195076 10.1007/s00262-006-0274-z PMC11031027

[32] Ichii S , Imai Y , Irimura T . Initial steps in lymph node metastasis formation in an experimental system: possible involvement of recognition by macrophage C‐type lectins. Cancer Immunol Immunother 2000; 49: 1–9. 10782861 10.1007/s002620050021 PMC11036940

[33] Napoletano C , Rughetti A , Agervig Tarp MP , *et al*. Tumor‐associated Tn‐MUC1 glycoform is internalized through the macrophage galactose‐type C‐type lectin and delivered to the HLA class I and II compartments in dendritic cells. Cancer Res 2007; 67: 8358–8367. 17804752 10.1158/0008-5472.CAN-07-1035

[34] Rodriguez T , Pacheco‐Fernández T , Vázquez‐Mendoza A , *et al*. MGL1 receptor plays a key role in the control of *T. cruzi* infection by increasing macrophage activation through modulation of ERK1/2, c‐Jun, NF‐κB and NLRP3 pathways. Cells 2020; 9: 51. 10.3390/cells9010108 PMC7017267 31906385

[35] Wan Y , Adair K , Herrmann A , *et al*. C1GalT1 expression reciprocally controls tumour cell–cell and tumour–macrophage interactions mediated by galectin‐3 and MGL with double impact on cancer development and progression. Cell Death Dis 2023; 14: 547. 37612278 10.1038/s41419-023-06082-7 PMC10447578

[36] Veglia F , Sanseviero E , Gabrilovich DI . Myeloid‐derived suppressor cells in the era of increasing myeloid cell diversity. Nat Rev Immunol 2021; 21: 485–498. 33526920 10.1038/s41577-020-00490-y PMC7849958

[37] van Vliet SJ , Gringhuis SI , Geijtenbeek TB , van Kooyk Y . Regulation of effector T cells by antigen‐presenting cells via interaction of the C‐type lectin MGL with CD45. Nat Immunol 2006; 7: 1200–1208. 16998493 10.1038/ni1390

[38] Nakamura K , Smyth MJ . Myeloid immunosuppression and immune checkpoints in the tumor microenvironment. Cell Mol Immunol 2020; 17: 1–12. 31611651 10.1038/s41423-019-0306-1 PMC6952382

[39] Gabrilovich DI . Myeloid‐derived suppressor cells. *Cancer* . Immunol Res 2017; 5: 3–8. 10.1158/2326-6066.CIR-16-0297 PMC5426480 28052991

[40] Li W , Ke C , Yang C , *et al*. LncRNA DICER1‐AS1 promotes colorectal cancer progression by activating the MAPK/ERK signaling pathway through sponging miR‐650. Cancer Med 2023; 12: 8351–8366. 36708020 10.1002/cam4.5550 PMC10134332

[41] Onami TM , Lin MY , Page DM , *et al*. Generation of mice deficient for macrophage galactose‐ and N‐acetylgalactosamine‐specific lectin: limited role in lymphoid and erythroid homeostasis and evidence for multiple lectins. Mol Cell Biol 2002; 22: 5173–5181. 12077344 10.1128/MCB.22.14.5173-5181.2002 PMC139776

[42] Laird PW , Zijderveld A , Linders K , Rudnicki MA , Jaenisch R , Berns A . Simplified mammalian DNA isolation procedure. Nucleic Acids Res 1991; 19: 4293. 1870982 10.1093/nar/19.15.4293 PMC328579

[43] Neufert C , Becker C , Neurath MF . An inducible mouse model of colon carcinogenesis for the analysis of sporadic and inflammation‐driven tumor progression. Nat Protoc 2007; 2: 1998–2004. 17703211 10.1038/nprot.2007.279

[44] Yoshihara K , Yajima T , Kubo C , Yoshikai Y . Role of interleukin 15 in colitis induced by dextran sulphate sodium in mice. Gut 2006; 55: 334–341. 16162679 10.1136/gut.2005.076000 PMC1856088

[45] Terrazas CA , Huitron E , Vazquez A , *et al*. MIF synergizes with Trypanosoma cruzi antigens to promote efficient dendritic cell maturation and IL‐12 production via p38 MAPK. Int J Biol Sci 2011; 7: 1298–1310. 22110382 10.7150/ijbs.7.1298 PMC3221366

[46] Ogasawara N , Poposki JA , Klingler AI , *et al*. Role of RANK‐L as a potential inducer of ILC2‐mediated type 2 inflammation in chronic rhinosinusitis with nasal polyps. Mucosal Immunol 2020; 13: 86–95. 31641233 10.1038/s41385-019-0215-8 PMC6917894

