# Supplementary material for: The macrophage galactose‐type C‐type lectin 1 receptor plays a major role in mediating colitis‐associated colorectal cancer malignancy
Source: Immunol Cell Biol. 2025 Mar 3;103(5):444–60. doi: 10.1111/imcb.70011 (PMC12108702; doi:10.1111/imcb.70011)
Supplement: Supplementary file 1 — Supplementary figure 1. Supplementary figure 2. Supplementary figure 3. Supplementary figure 4. Supplementary figure 5. Supplementary table 1. [file IMCB-103-444-s001.pdf]

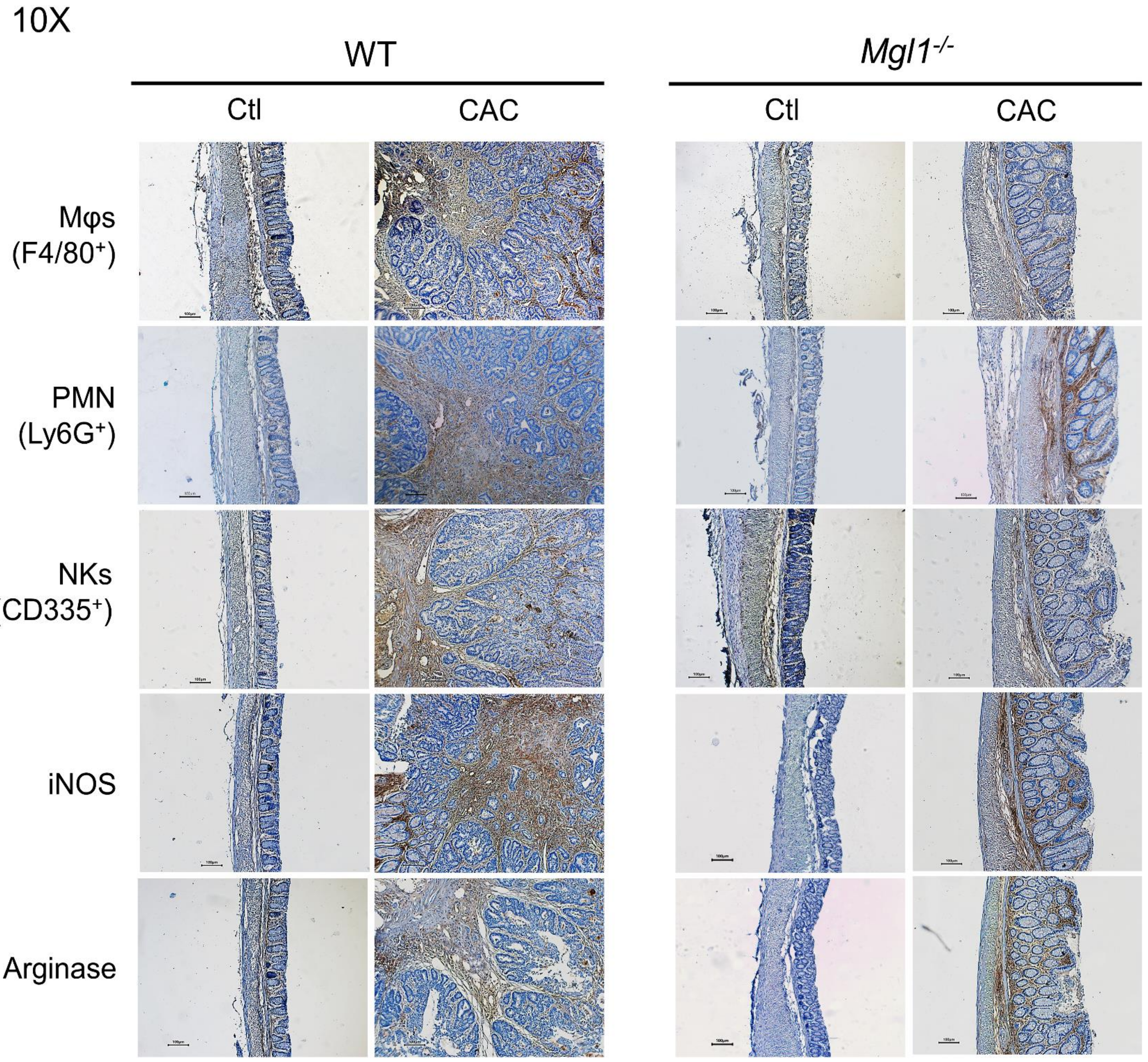

Supplementary figure 1: mMGL1 deficiency decreases the abundance of Mφs, PMN cells, NK cells, iNOS and arginase in tumor infiltration. The presence of Mφs (F4/80$^+$), PMN (Ly6G$^+$), NK cells (CD335$^+$), iNOS and arginase in the colons of *Mgl1*$^{-/-}$ and WT mice was assessed using immunohistochemistry. Representative immunostaining images of the descending colon were captured at 10X magnification. The data are representative of three independent experiments (n = 3).

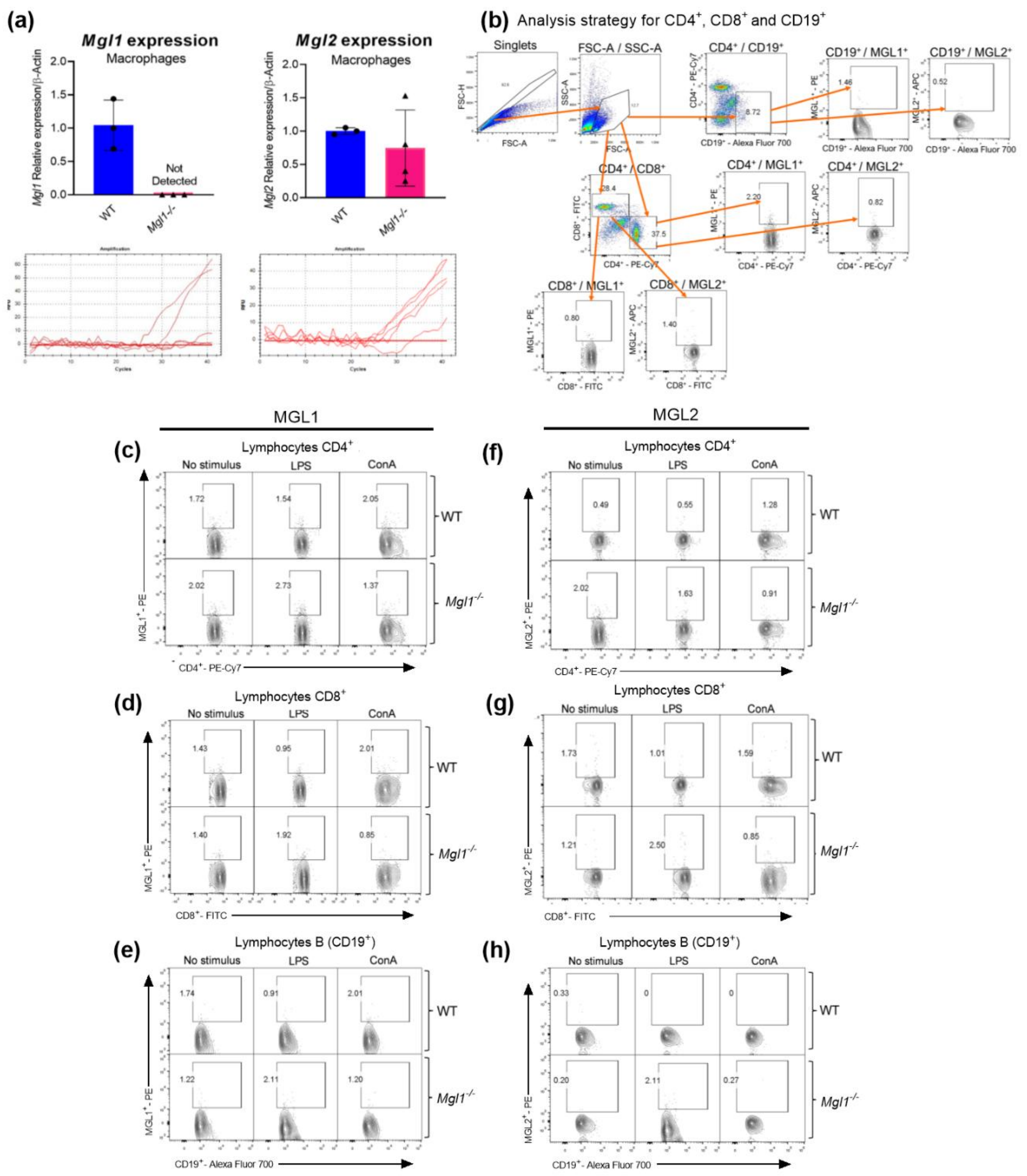

Supplementary figure 2: MGL1and MGL2 is expressed in macrophages but not in B and T cells. (a) mRNA expression of *Mgl1* and *Mgl2* in macrophages differentiated from bone marrow of WT and *Mgl1*$^{-/-}$ mice measured by RT-PCR. **(b)** Gating strategy used to identify CD4^{+}, CD8^{+} T cells and the CD19^{+} B cell populations, as well as the expression of MGL1/2. Representative plots of the expression of MGL1 in naïve **(c)** CD4^{+} lymphocytes; **(d)** CD8^{+} lymphocytes; **(e)** B (CD19^{+}) lymphocytes, and the expression of MGL2 in naïve **(f)** CD4^{+}; **(g)** CD8^{+} and **(h)** B (CD19^{+}) lymphocytes without or with LPS (100 ng/mL) and ConA (1µg/ml) stimulation are presented. The data are representative of three independent experiments (n = 3).

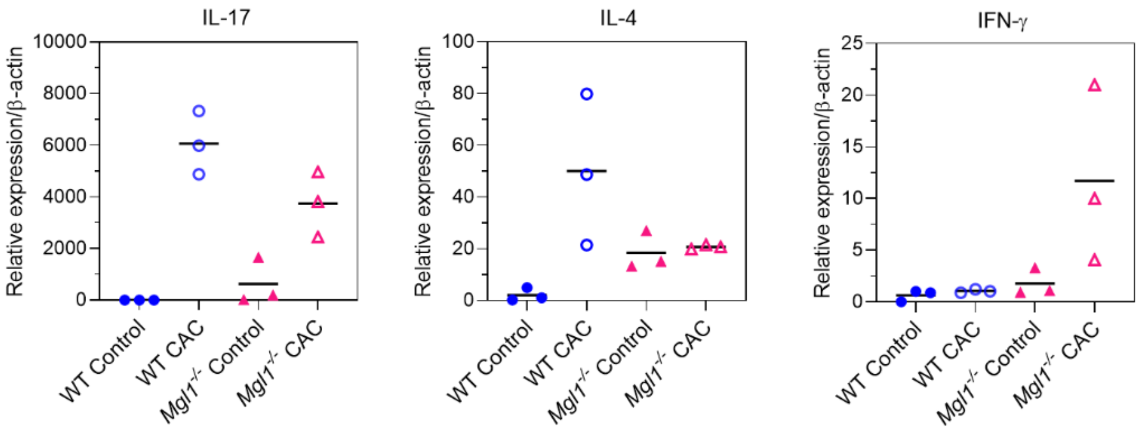

Supplementary figure 3: *Mgl1$^{-/-}$* CAC mice exhibit lower IL-17 and IL-4 expression but enhanced IFN-γ expression in colon tissue. WT and *Mgl1$^{-/-}$* colon tissues were removed 75 days post-CAC induction. The relative expression of the transcripts of IL-4, IL-17, and IFN-γ normalized to the constitutive expression of β-actin, was measured in total cDNA synthesized from total RNA extracted and purified from healthy control tissue and tumors by quantitative real-time polymerase chain reaction (qRT-PCR). The graphical data represents three independent experiments that are presented as the means ± standard errors of the means (n = 3 mice per group). Statistical analysis was not performed because the "n" was insufficient.

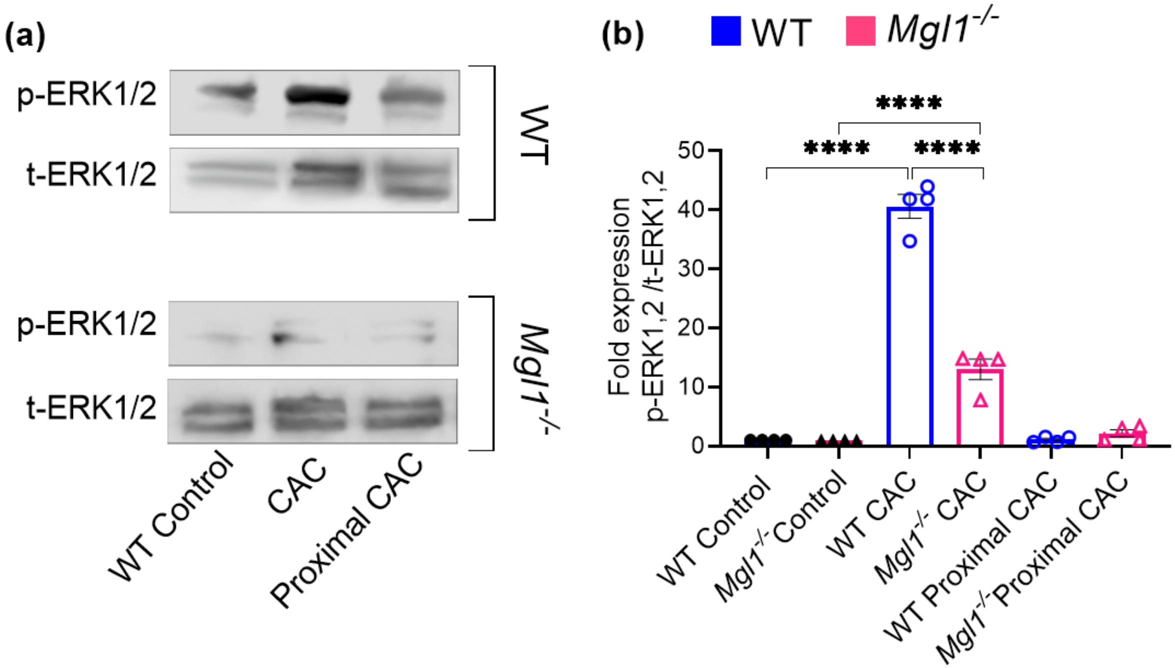

Supplementary figure 4: *Mgl1*$^{-/-}$ CAC mice exhibit reduced activation of the ERK1/2 signalling pathway in colon tissue. **(a)** WT and *Mgl1*$^{-/-}$ colon tissues were removed 75 days post-CAC induction. ERK1/2 phosphorylation levels were measured in total protein lysates from healthy control groups and from colonic tumours and proximal colon tissue (tissue without visible tumours) from CAC mice was measured using western blot. Colon tissue and tumours were macerated and lysed to obtain protein. The membranes were incubated with the following primary antibodies: p44/42 MAPK (ERK1/2), p-p44/42, and MAPK (p-ERK1/2) (Cell Signaling, Danvers, MA, USA). **(b)** Fold change in ERK1/2 expression. The graphical data represent three independent experiments and are presented as the means $\pm$ standard errors of the means (n = 3 - 5 mice per group). Statistically significant differences between groups were assessed using one-way ANOVA with Tukey's multiple comparisons test. Significance is indicated as $^{****}P < 0.0001$. GraphPad Prism 8.3 software was used.

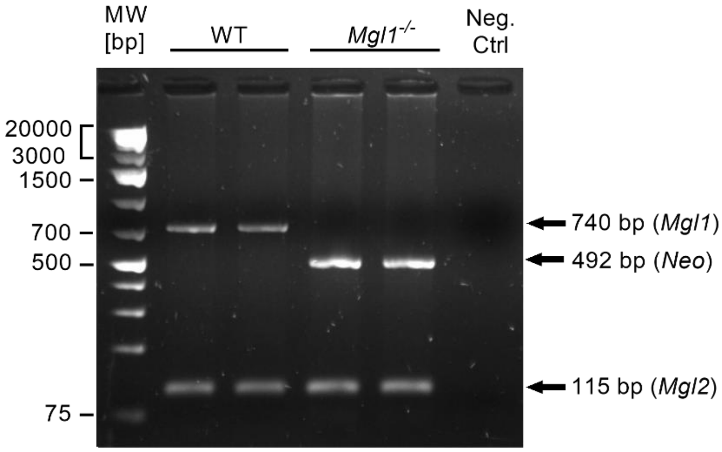

Supplementary figure 5: Genotyping of *Mgl1*$^{-/-}$ and WT mice. Genotyping was routinely performed on DNA isolated from the tail snips of WT and *Mgl1*$^{-/-}$ mice. PCR for the amplification of the *Mgl1, Mgl2* and *Neo* genes was performed with Taq DNA polymerase (Ampliqon, Bioreagents and Molecular Diagnostics, Denmark) following the manufacturer's instructions. The PCR products were analysed using electrophoresis on a 1.5% agarose gel and viewed under UV light (Bio-Rad, Hercules, CA, USA). MW: molecular DNA marker; WT: wild type homozygous genotype; *Mgl1*$^{-/-}$: *Mgl1* knockout homozygous genotype, *Mgl1* sequence interrupted by the neomycin gene; Neg. Ctrl: negative control. Primers used were described in Supplementary table **1**.

Supplementary table 1: Primer sequences used for PCR analysis. All primers were synthesized by Sigma–Aldrich, Mexico City, Mexico.

Primer	Sequence	TM (°C)	Amplicon size (bp)
mgl1 F	5′-CTT GGT CCC AGA TCC GTA TC-3′	54	715
mgl1 R	5′-ATG TCA TGA CTC AGG ATC-3′		
mgl2 F	5’-CTT TTC TTA CGA TGG TCG TTG GA-3′	58	115
mgl2 R	5’-TGA GGC TAT AAG TTG TGG GGA-3′		
neo F	5′-AGG ATC TCC TGT CAT CTC ACC TTG CTC CTG-3′	65	492
neo R	5′-AAG AAC TCG TCA AGA AGG CGA TAG AAG GCG-3′		
Il-17 F	5’-AAA GCT CAG CGT GTC CAA AC-3’	59	129
Il-17 R	5’-TGG AAC GGT TGA GGT AGT CTG-3’		
Il-4 F	5’-CGA AGA ACA CCA CAG AGA GTG AGC T-3’	58	181
Il-4 R	5’-GAC TCA TTC ATG GTG CAG CTT ATC G-3’		
Ifn-γ F	5’-AGC GGC TGA CTG AAC TCA GAT TGT AG-3’	57	247
Ifn-γ R	5’- GTC ACA GTT TTC AGC TGT ATA GGG-3’		
β-actin F	5’-GTG ACG TTG ACA TCC GTA AAG A-3′	60	225
β-actin R	5’-GCC GGA CTC ATC GTA CTC C-3′		
